# The community‐wide effectiveness of municipal larval control programs for West Nile virus risk reduction in Co nnecticut, USA

**DOI:** 10.1002/ps.6559

**Published:** 2021-08-05

**Authors:** Joseph R McMillan, Christina A Harden, James C Burtis, Mallery I Breban, John J Shepard, Tanya A Petruff, Michael J Misencik, Angela B Bransfield, Joseph D Poggi, Laura C Harrington, Theodore G Andreadis, Philip M Armstrong

**Affiliations:** ^1^ The Connecticut Agricultural Experiment Station New Haven CT USA; ^2^ The Northeast Regional Center of Excellence in Vector‐borne Diseases Cornell University Ithaca New York USA; ^3^ Pennsylvania State University, State College University Park PA USA; ^4^ Division of Vector‐borne Diseases Centers for Disease Control and Prevention Fort Collins CO USA; ^5^ Yale University New Haven CT USA; ^6^ Cornell University Ithaca NY USA

**Keywords:** mosquito larval control, West Nile virus, *Culex pipiens* complex, catch basin

## Abstract

**BACKGROUND:**

Mosquito larval control through the use of insecticides is the most common strategy for suppressing West Nile virus (WNV) vector populations in Connecticut (CT), USA. To evaluate the ability of larval control to reduce entomological risk metrics associated with WNV, we performed WNV surveillance and assessments of municipal larvicide application programs in Milford and Stratford, CT in 2019 and 2020. Each town treated catch basins and nonbasin habitats (Milford only) with biopesticide products during both WNV transmission seasons. Adult mosquitoes were collected weekly with gravid and CO_2_‐baited light traps and tested for WNV; larvae and pupae were sampled weekly from basins within 500 m of trapping sites, and *Culex pipiens* larval mortality was determined with laboratory bioassays of catch basin water samples.

**RESULTS:**

Declines in 4th instar larvae and pupae were observed in catch basins up to 2‐week post‐treatment, and we detected a positive relationship between adult female *C. pipiens* collections in gravid traps and pupal abundance in basins. We also detected a significant difference in total light trap collections between the two towns. Despite these findings, *C. pipiens* adult collections and WNV mosquito infection prevalence in gravid traps were similar between towns.

**CONCLUSION:**

Larvicide applications reduced pupal abundance and the prevalence of host‐seeking adults with no detectable impact on entomological risk metrics for WNV. Further research is needed to better determine the level of mosquito larval control required to reduce WNV transmission risk.

## Graphical abstract

Town‐wide larval control reduced the number of larvae in catch basins, yet had little impact on adult mosquito abundance and West Nile virus infection prevalence.

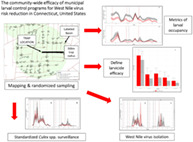

© 2021 The Authors. *Pest Management Science* published by John Wiley & Sons Ltd on behalf of Society of Chemical Industry.

## 1 INTRODUCTION

West Nile virus (WNV) is a mosquito‐borne virus (i.e. arbovirus) transmitted by *Culex* spp. mosquitoes among birds.^1^ The virus was first discovered in the USA in 1999 and spread throughout North and South America where it continues to be a threat to human and wildlife health.^1^, ^2^, ^3^ The intensity and frequency of WNV epidemics in the USA varies among regions and years, although WNV accounts for the majority of reported arbovirus infections in humans.^2^, ^3^ There are few economic incentives to developing a human WNV vaccine,^4^ and prevention of WNV exposure relies on Integrated Vector Management (IVM).^5^ IVM strategies recommend multiple measures, including personal protection and adult/larval source reduction/removal methods, in order to control vector populations and reduce risk of arboviral exposures. For WNV, mosquito control efforts are led by a variety of local to regional‐level authorities. Control targets range from larvicidal treatments in roadside catch basins, to removal of standing water from containers on public and private properties, to aerial and ground‐level applications of insecticides during WNV public health emergencies.^5^

Certain regions of the USA maintain well‐funded and publicly supported mosquito control operations. Throughout the remainder of the USA, mosquito control for WNV is the responsibility of local authorities, many of which lack the resources and support to implement any mosquito control strategy(s).^6^, ^7^ Local authorities that do implement mosquito control strategies commonly utilize dual approaches including public health messaging and larval source reduction with insecticides.^8^ Larvicide treatments by such authorities are limited to properties under their jurisdiction,^7^, ^9^ and the primary targets for larviciding include roadside catch basins, storm drains and retention ponds. Catch basins are below street‐level structures, which facilitate precipitation run‐off into sewage and waste water treatment systems. Their design includes a sump at their base below the outflow line meant to capture and prevent large debris from entering water treatment systems. Sumps can contain eutrophic water for extended periods of time making them ideal larval habitats for the primary urban mosquito vectors of WNV in the USA, *Culex pipiens* complex.^10^, ^11^, ^12^ Other sources of *C. pipiens* complex larvae have been documented in urban environments,^13^, ^14^ but these habitats are more difficult to identify and treat than catch basins. Thus, larval control in catch basins represents a common and widely replicated approach to WNV control in the US.

Operational aspects of *C. pipiens* control in catch basins are well‐documented; studies have examined the influence of land cover and climate on the abundance of larvae,^15^, ^16^ the effectiveness and residual activity of larvicide products,^17^, ^18^, ^19^, ^20^, ^21^, ^22^ and the physical properties that facilitate larval development and control failures.^23^, ^24^, ^25^ There is less evidence on the capability of larval control in catch basins and other larval habitats to reduce WNV risk metrics.^26^, ^27^, ^28^ To examine the ability of municipal larval control programs to affect WNV transmission, we performed WNV mosquito surveillance coupled with systematic evaluations of larvicide treatments in catch basins in two towns in CT, USA with historical evidence of enzootic WNV transmission yet which utilize different mosquito control approaches. The objectives of our study were to assess the effectiveness of larvicides in catch basins, compare catch basin and surveillance collections between the towns, and examine whether catch basin collections were predictive of WNV infection metrics in captured adult mosquitoes. Our results address a critical need for applied epidemiological research into public health interventions for WNV at spatial scales relevant to local governments.

## 2 MATERIALS AND METHODS

### 2.1 Surveillance site characteristics

**Figure 1:**
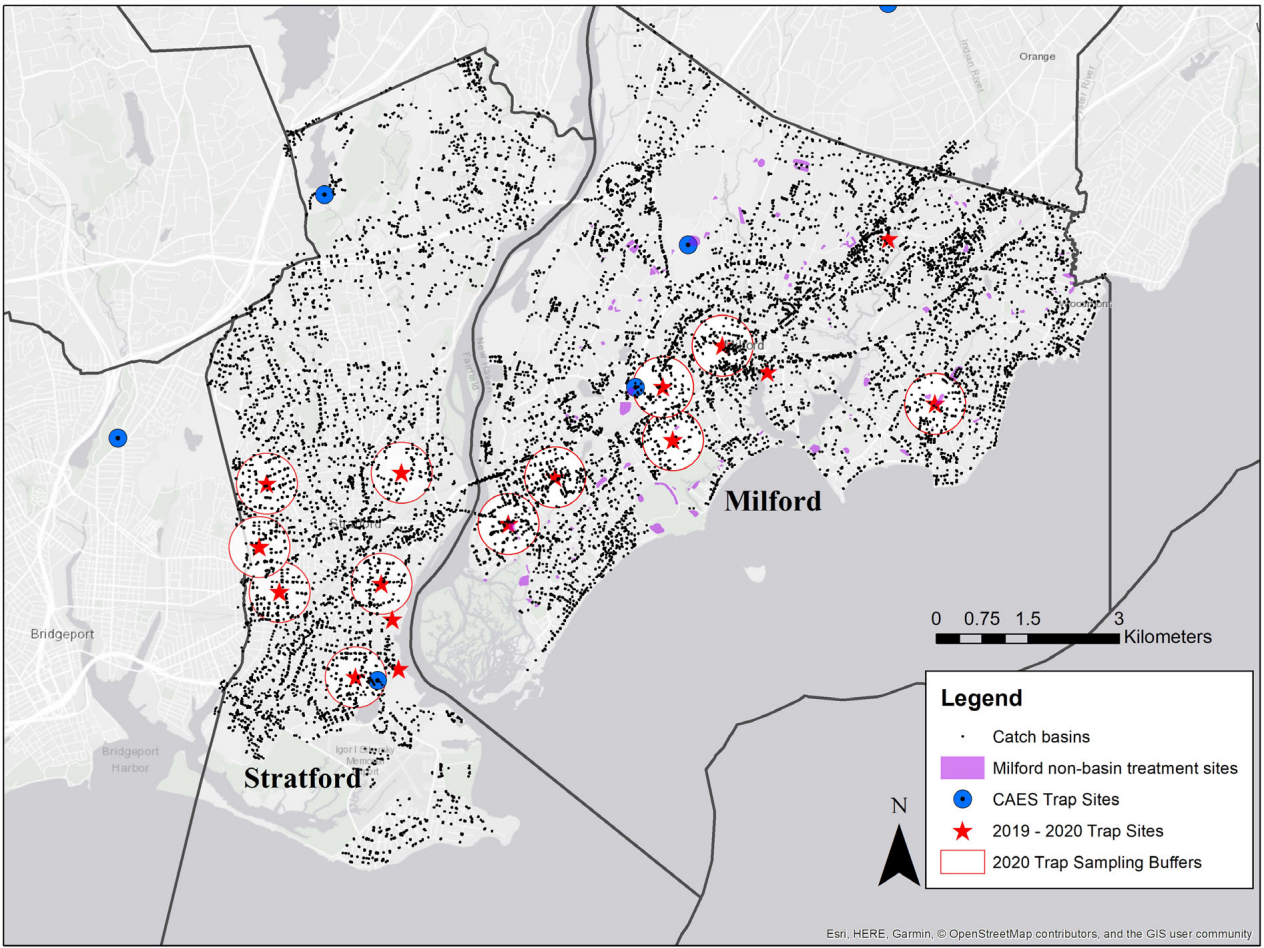
Map of surveillance and catch basin sampling sites in Milford and Stratford, CT 2019–2020. Red stars, adult mosquito and West Nile virus surveillance sites; white with red border circles, 500 m sampling buffer instituted in 2020; small black points, catch basin locations; purple regions, nonbasin larvicide treatment areas in Stratford; solid black lines, each townʼs political boundary with town names listed in bold, black text. The Connecticut Agricultural Experiment Stationʼs statewide mosquito and arbovirus surveillance locations in the region are shown for reference using blue circles with black dots. The figure was produced in ArcMap 10.5.1 (ESRI) using the OpenStreetsMap World Base and Reference maps.

Mosquito control in CT for nuisance and disease vectors is the responsibility of local municipalities (with the exception of state‐owned property), and there are no centralized mosquito abatement/control districts in CT. Thus, each town implements their own program pursuant of budgetary limitations and public support. Our study design therefore focused on surveillance of mosquitoes and larvicide effectiveness in towns that utilize different approaches to mosquito control. After consulting with mosquito control applicators and public health partners, we implemented our study in Milford and Stratford, CT during the 2019 and 2020 WNV transmission seasons (June–October). Milford and Stratford are neighboring towns located in the southwest portion of CT, with Milford located directly east and Stratford directly west of the mouth of the Housatonic River (Fig. 1). Milford has a larger incorporated area (Milford 65.1 km^2^; Stratford 51.5 km^2^), whereas Stratford has a higher population density (Milford 841 persons km^–2^; Stratford 1007 km^–2^) (2019 US Census population estimates). The Connecticut Agricultural Experiment Station (CAES) maintains two mosquito and arbovirus surveillance sites in each town, and each townʼs southern surveillance site is considered at high risk for WNV^29^ (Fig. 1). However, the southern Stratford site tends to collect more *C. pipiens* and detect more WNV‐positive samples than Milfordʼs southern site (https://portal.ct.gov/CAES/Mosquito-Testing/Introductory/State-of-Connecticut-Mosquito-Trapping-and-Arbovirus-Testing-Program).

Each town implements a different approach to mosquito control, although control of mosquitoes in catch basins for WNV risk prevention is central to each townʼs approach. *S*‐methoprene, an insect‐growth regulator that is the main active ingredient in many mosquito larval control products, is restricted in CT as well as in other regions of the northeast USA, so biopesticides are the most commonly used products for larval control; the products utilized in this study were at the discretion of the applicator. Milford contracts with a private applicator for a comprehensive program which consists of biweekly treatments of tidal and nontidal/flood‐prone habitats with *Bacillus thuringiensis israelensis* and *Lysinibacillus sphaericus* products as well as catch basin treatments with *L. sphaericus* (applied as ≈20 g Vectolex FG) during the summer months. The nonbasin habitats in Milford are treated from April/May to September/October, and control of nuisance mosquitoes also can include home evaluations when requested by a resident. The basin‐specific applications in Milford are for control of *Culex* spp. mosquitoes and WNV, and applications are performed in approximately June, July and August (≈8000 basins, 123.6 basin km^–2^). The same mosquito control applicator contracts for control in Stratford (≈5000 basins, 98.6 basins km^–2^); however, Stratford has fewer resources for control and only contracts for a single treatment of catch basins with the same *L. sphaericus* product used in Milford. The timing of the single Stratford treatment varies between years and some years are not funded as a consequence of budgetary constraints. Funds were made available for the single treatments in 2019 and 2020 in response to a statewide WNV epidemic in 2018 that resulted in 22 human cases (the most in a single year in CT). See Table 1 for a listing of basin and nonbasin treatments in Milford during each year of the study. All surveillance site‐specific maps of catch basin locations and IDs are provided as Supporting Information Figures S1–S14 .

**Table 1:** Mosquito larval control application summaries in Milford, CT 2019–2020

		2019					2020				
Month	Route	Visits	Applications	Product	Area treated (km^2^)	Amount product (kg)	Visits	Applications	Product	Space Treated (km^2^)	Amount product (kg)
April	A	None applied					44	2	Vectobac G	0.0081	1.81
									Vectobac GS	0.0081	1.81
	B	None applied					42	17	Vectobac G	0.081	23.6
									Vectobac GS	0.081	23.6
May	A	None applied					88	4	Vectobac G	0.02	4.99
									Vectobac GS	0.02	4.99
	B	82	38	Aquabac	0.088	27.67	7.71 7.71	7	Vectobac G	0.028	7.71
				Vectolex FG	0.094	46.49			Vectobac GS	0.028	7.71
June	A	88	39	Spheratex	0.0089	10.89	44	0	None applied		
				Vectobac G	0.10	27.22					
				Vectolex FG	0.0008	2.72					
	B	95	55	Spheratex	0.0008	1.81	42	2	Vectobac G	0.0081	1.36
				Vectobac G	0.094	23.59			Vectobac GS	0.0081	1.36
				Vectobac G/GS	0.16	46.72			Vectolex FG	0.004	0.23
	Basins*	6216		Vectolex FG		151.05	7576		Vectolex FG		73.0
July	A	88	30	Vectobac G	0.067	18.14	88	7	Vectobac G	0.049	6.8
				Vectobac G/GS	0.15	65.32			Vectobac GS	0.049	6.8
				Vectobac GS	0.012	7.03			Vectolex FG	0.013	1.13
				Vectolex FG	0.0016	3.18					
	B	86	39	Vectobac G	0.069	24.29	84	5	Vectobac G	0.0061	1.36
				Vectobac G/GS	0.13	59.87			Vectobac GS	0.0061	1.36
				Vectolex FG	0.003	5.90			Vectolex FG	0.003	0.18
	Basins*	7576		Vectolex FG		151.05	7127		Vectolex FG		65.3
August	A	88	29	Vectobac G	0.057	12.7	88	11	Vectobac G	0.022	3.4
				Vectobac G/GS	0.047	20.87			Vectobac G/GS	0.032	8.16
				Vectolex FG	0.0042	6.35			Vectobac GS	0.022	3.4
									Vectolex FG	0.012	1.13
	B	96	33	Vectobac G	0.13	29.03	84	5	Vectobac G	0.0081	2.04
				Vectobac G/GS	0.036	10.89			Vectobac G/GS	0.010	0.95
				Vectolex FG	0.0053	10.89			Vectobac GS	0.004	1.13
	Basins*	7576		VectoLex FG	151.05		7127		Vectolex FG		56.7
September	A	69	24	Vectobac G	0.045	16.56	75	6	Vectobac GS	0.004	0.45
				Vectobac GS	0.0085	5.90			Vectobac G/GS	0.024	10.5
				Vectolex FG	0.0049	5.44					
	B	76	23	Vectobac G	0.012	3.18	55	3	Vectobac G	0.01	1.36
				Vectolex FG	0.0077	7.80			Vectobac G/GS	0.004	2.72
									Vectobac GS	0.01	1.36
October	A	21	4	Vectobac G	0.0040	0.45	None applied				
				Vectolex FG	0.0012	0.95					

^*^ Specific application dates: 2019, 20–22 May, 28 June–5 July, 29–31 July; 2020, 2–4 June, 13–15 July, 19–24 August.

### 2.2 Field surveillance 2019

#### 2.2.1 WNV surveillance

In April and May 2019, eight surveillance sites in each town were chosen for WNV surveillance (Figs 1 and S1). These sites all were located in an urban setting near the I‐95 interstate corridor (a region considered at greater risk of WNV in CT) and surrounded by catch basins accessible for sampling. Eight sites per town represented the maximum amount of sampling effort we could sustain at a preferred weekly sampling interval. Each town was separated into routes, with Milford consisting of an E–W route and Stratford consisting of a N–S route; traps in these routes always were sampled at the same time and the density of traps within each route minimized possible pseudo‐replication of collections that may occur when using a higher density of traps.^30^, ^31^ Adult female mosquitoes were sampled at all surveillance sites between 10 June and 31 October with a single gravid trap baited with a lactalbumin‐hay‐yeast infusion^32^ and a single ground‐level CO_2_‐baited light trap. Each trap was set in the afternoon and collected the following morning. All gravid and light trap collections were returned to Connecticut Agricultural Experiment Station (CAES) where female mosquitoes were sorted from by‐catch and stored at −80 °C until specimens could be identified to species morphologically using a dichotomous key^33^ and tested for WNV in pool sizes ≤50 individuals following published cell culture and reverse transcription polymerase chain reaction (RT‐PCR) methods.^34^, ^35^

#### 2.2.2 Catch basin sampling

In May and June, catch basins near four surveillance sites in each town were surveyed for the presence of water and the ability to remove the catch basinʼs grate. Sample basin determination was not randomized, but was based on the level of traffic and the ease/safety of sampling. Six to 10 basins per surveillance site were chosen for weekly sampling within 24 h of trap setting. At each basin, if water was present at the time of sampling, the grate was removed, three dips were performed with a 500‐mL plastic Nasco extended sampler, and the presence of *Culex* spp. egg rafts, any instar of larvae, and pupae in any of the dips was recorded. Recording presence/absence of larvae and pupae is a common field check of larvicide products such as VectoLex FG,^36^ and this was considered a sufficient field measure of larvicide effectiveness. We also collected a 300‐mL water sample from the basin using Nasco Sampling Line fitted with a 532‐mL Whirl‐Pak bag (Nasco, Fort Atkins, WI, USA).

#### 2.2.3 Larvicide bioassay

In order to better define the effectiveness of *L. sphaericus* in treated basins, we tested water samples from a subset of basins for realized mortality using CAES' *C. pipiens* laboratory colony. All water samples were returned to CAES and stored in a 4 °C refrigerator until tested. Every week, two to three *C. pipiens* egg rafts were placed in 2 L water in enamel pans and held at 24°C at 80% humidity. Larvae were fed with an addition 5–10 mL of a 3:2 bakers' yeast/ liver powder solution every other day. For the bioassays, 100 mL of a catch basinʼs water sample was filtered through a paper coffee filter into a 300‐mL disposable plastic cup and 15 3rd–4th instar *C. pipiens* were inserted into the water. We ran a positive and negative control for every ten water samples tested: positive controls were inoculated with the LC95 for *L. sphaericus* ,^37^ whereas negative controls contained just larvae and water. All samples and controls received <0.05 g TetraMin (Tetra, Blacksburg, VA, USA) fish food at the beginning of every assay. Larval mortality was defined following Burtis *et al*. 2020^37^ and counted 24 h after larvae were inserted in the water sample. All mortality results were corrected for mortality in the negative control using an Abbot Correction [(% sample mortality–% control mortality)/(100–% control mortality)] * 100. All water samples were tested within 7 days of collection.

### 2.3 Field surveillance 2020

All mosquito and WNV surveillance locations and methods remained the same in 2020, and surveillance methods took place between 1 June and 16 October. We made significant changes to our methodology following a series of statistical power analyses using preliminary data from the 2019 portion of the study (see SI 2 for the R‐code to reproduce these analyses). Our primary tests included determining: realized statistical power in 2019 (defined as the proportion of model simulations of Milford *versus* Stratford collections with *P*‐values <0.05),^38^ the number of surveillance sites needed to detect a 20% difference in gravid trap collections (which was the observed difference in collections in 2019), the number of catch basins to sample in order to link collections in catch basins to collections in gravid/light traps (based on the 2019 metric of presence/absence of larvae/pupae), and whether other quantitative metrics from catch basins could predict trap collections. We followed simulation recommendations for generalized linear mixed effects models (GLMMs) developed by Johnson *et al*. 2015.^38^ Simulation data were generated using the seasonal average of trap collections under a negative binomial distribution and/or the prevalence of pupae in catch basins in each town under a binomial distribution using the glmmmisc and lme4 R packages.^38^, ^39^

#### 2.3.1 Catch basin sampling

Initial power analyses suggested only marginal increases in statistical power by increasing the number of traps in each town. Therefore, we developed a strategy to collect quantitative metrics of larval and pupal occupancy in 10% of basins within 500 m of each surveillance site. Based on preliminary analyses with various buffer sizes and our experience sampling basins in 2019, the 500 m buffer and 10% sample size represented a distance at which we could maximize our weekly sampling of a subset of basins (the 10% threshold resulted in between 10 and 20 basins per surveillance site). The 500 m buffer also represents a conservative estimate of mosquito flight distances reported for *Culex* spp. and other mosquitoes.^40^, ^41^, ^42^

We obtained GIS files of all catch basin locations in Milford and Stratford from each townʼs respective public works department. In arcmap v10.5.1 (ESRI) we drew 500 m buffers around each trap site to create a new layer with the number and location of basins within the buffer. Basins were assigned a random identification number, IDs were sorted in ascending order, and basins were evaluated sequentially until the 10% threshold was met. Basins were initially eliminated if they were not located on a road likely to be treated by the applicator (e.g. interstates). Basins then were assessed in the field for the presence of water, the ability of our sampling equipment to fit through the basinʼs grate, and whether sampling the basin would pose any safety risks. Evaluations took place between 1 May and 2 July and some sites required multiple evaluations to achieve the 10% sampling target due to drought‐like conditions. Two surveillance sites in each town were excluded from these evaluations: one Milford site contained a majority of basins located along a major throughway as well as on private property within the 500 m buffer, one Milford site contained a majority of natural spaces and open water within the 500 m buffer, and two sites in Stratford shared a large degree of overlap between each siteʼs 500 m buffer. Catch basin selection summaries for 2020 are available in Table S1.

We built a larval sampler and a water sampler, each capable of slipping through standard catch basin grates (a square or rectangular grid with spacing roughly 63.5 mm). The larval sampler was a 101.5 mm rectangular aquarium fish net (quick‐net™) modified to slip though the catch basin grate and duct‐taped to a 1.2 m long, 12.7 mm diameter PVC pipe. The PVC pipe included a fitting so that an additional 1.2 m PVC segment could be added to the sampler in order to sample catch basins >2 m deep. To sample larvae and pupae, a figure‐8 sweep was conducted through the surface of the water column, ensuring that the sweep extended through the middle to the walls of the basin. The net then was inverted and larvae and pupae were washed out of the net with ≈300–500 mL tap water into a 228.6 × 228.6 mm rimmed white plastic pan. If pupae and/or 4th instar larvae were noted in the sample, the sample was transferred to a 532‐mL Whirl‐Pak bag, the location and date of the sample was noted, and the sample was returned to CAES. The water sampler was constructed of a 177.8 mm piece of 38.1 mm diameter PVC piping sealed at one end with a 43.2 mm rubber stopper and all‐purpose silicone sealer. A hole was made near the other end of the pipe and a 4.5 m length of nylon rope was tied to the pipe. Six steel washers were tied to the string near the top of the sampler to ensure that the sampler sank when inserted into the basinʼs water column. When between zero to five pupae and/or 4th instar larvae were detected in a sweep sample, the water sampler was inserted into the water column to collect water, transferred to a 532‐mL Whirl‐Pak bag marked with the location and date of the sample, and the sample was returned to CAES. Separate larval and water samplers were used by each field technician in each town to reduce possible larvicidal contamination between sampling locations.

All 4th instar larvae were counted up to 100 per basin, preserved in 70% ethanol and identified to species following Andreadis *et al* 2005.^33^ All pupae were counted and separated into BioQuip Emergence Chambers by sample date and location, and pupae were held at 24.5 °C/80% relative humidity in a 16 h:8 h, light:dark photoperiod until emergence. Emerged adults were anesthetized for 60 min at −4 °C then identified to sex and species morphologically.^33^ All water samples were screened for mortality using the procedures described for 2019.

### 2.4 Statistical analyses

The objectives of our study were to assess the effectiveness of larvicides in treated catch basins, compare catch basin and adult mosquito collections between the two towns, and examine associations between *C. pipiens* immature collections in catch basins, and WNV and adult mosquito collections. Larvicide treatments were categorized as a five‐level term based on the timing of catch basin treatments; because town‐level treatments could take up to 5 days, the first to fourth week post‐treatment were considered the four levels of treatment. Any collections that took place either before the first seasonal application or five or more weeks post an application were considered a ‘No Treatment’ reference; this designation reflects the documented declining effectiveness of biopesticides in basin environments^28^, ^43^, ^44^, ^45^ and the listed maximum label duration of the VectoLex FG product. We chose to model larvicide treatments as a multilevel categorical term because the final form of our statistical analyses aggregated data to the level of a week. Daily temperature and precipitation records from the Sikorsky Memorial Airport in Stratford, CT were obtained through NOAAʼs Climate Data Online search tool (https://www.ncdc.noaa.gov/cdo-web/) and the weekly average for each variable was then calculated for each week of sampling. Weekly climate variables were assumed to be the same across all surveillance sites.

All data were analyzed in R v3.6.3.^46^ The final form of each analysis was a GLMM with crossed random intercept effects^47^ for each spatial (i.e. sampling location) and temporal (i.e. week of sampling) component of the data. Each response and predictor variable were examined individually using GLMMs. Response terms were modeled according to the form of the data: presence/absence and proportional data was modeled as a binomial distribution, count data were modeled as a Poisson‐ or negative binomial distribution, and minimum infection rates were first log+1 transformed and then modeled as a Gaussian distribution. Predictor terms in the catch basin specific analyses included Town, the larvicide treatment variable, and average weekly temperature and precipitation as fixed effects, and catch basin ID and week of collection as crossed random intercept effects. Predictor terms in the adult mosquito and WNV infection prevalence analyses included Town, the larvicide treatment variable, average weekly temperature and precipitation, and metrics of larval and pupal occupancy, as well as bioassay mortality estimates within 500 m of each surveillance site as fixed effects, and trapping location and week of collection as crossed random intercept effects. In the univariate analyses, each predictor showing a reduction in the Akaike Information Criteria (AIC) beyond a random effects‐only GLMM was selected for evaluation in a multi‐term GLMM. In each multi‐term GLMM, all predictor variables were evaluated using the ‘drop1’ function with a χ^2^‐test available in base R, and any variables that did not improve AIC were dropped from the GLMM. Interaction terms were only assessed once all additive terms were determined; no three‐way interactions were considered.

All GLMMs were coded, evaluated and plotted using a combination of the glmmtmb, ggeffects, sjplot and ggplot2 R packages.^48^, ^49^, ^50^ Owing to methodological differences in 2019 and 2020, each year was considered a separate study and analyzed independently.

## 3 RESULTS

All GLMM tables are provided as Supporting Information Tables S2‐S13.

### 3.1 Field season 2019

**Figure 2:**
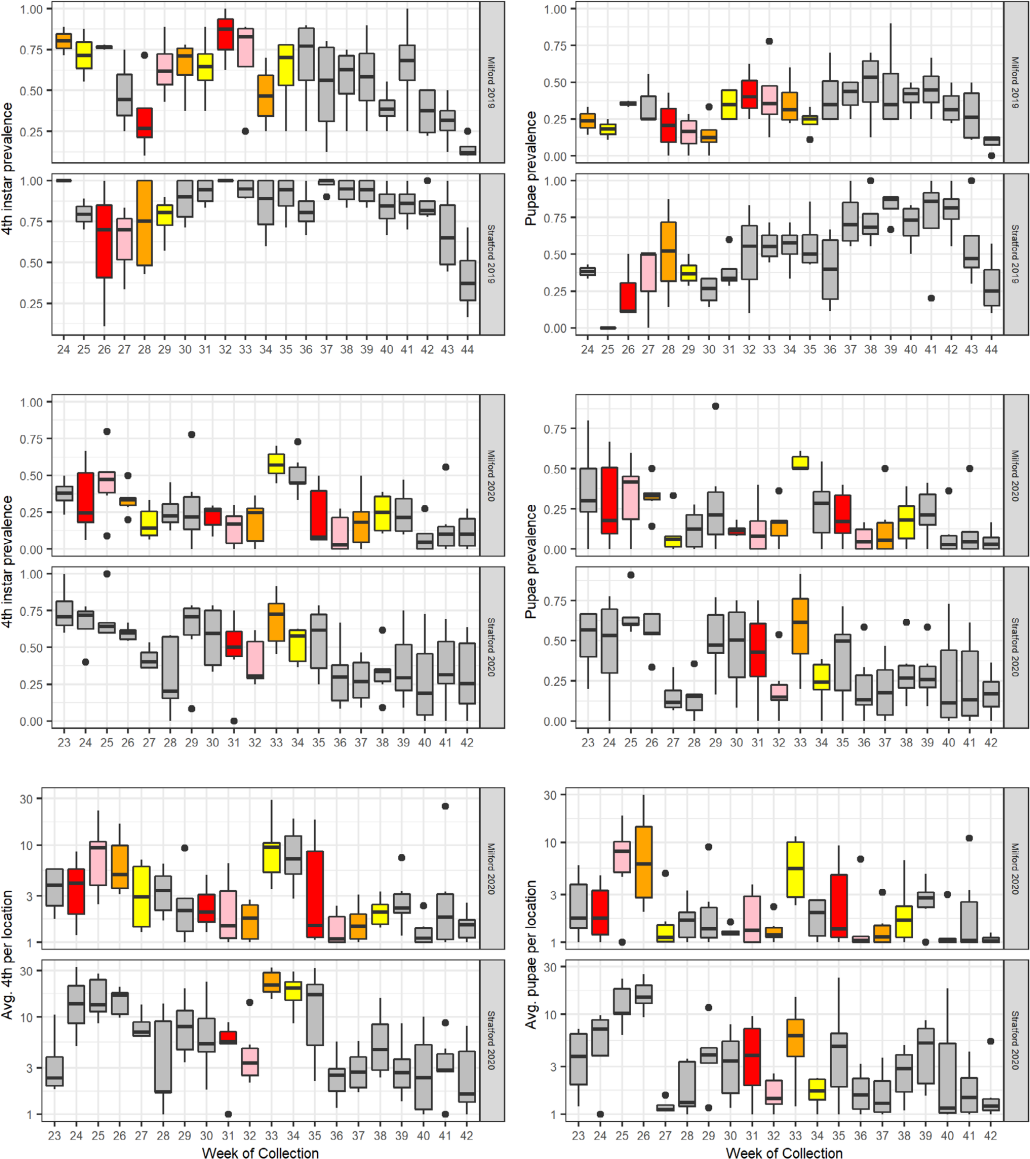
Weekly catch basin collections by town in 2019 and 2020. Colors indicate the larvicide treatment variable designation (red, week 1; pink, week 2; orange, week 3; yellow, week 4; grey, no or assumed decline of treatment effectiveness).

**Figure 3:**
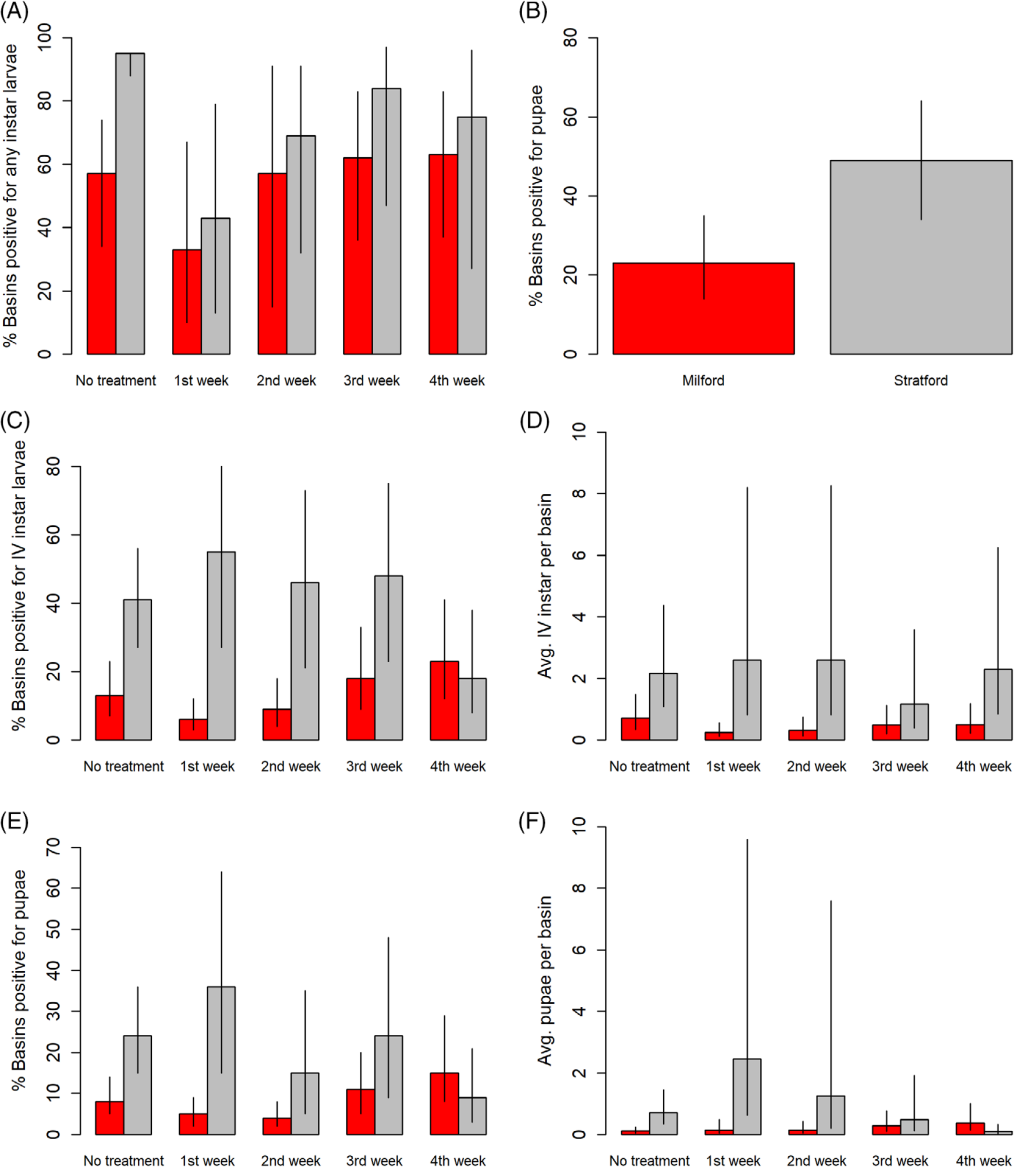
The predicted effect of the Town‐by‐Treatment interactions terms from GLMMs comparing catch basin collections by town in 2019 (A–B) and 2020 (C–F). Percentage of basins: (A) in 2019 positive for of any instar larvae, (B) in 2019 positive for pupae, (C) in 2020 positive for 4th instar larvae and (D) in 2020 positive for pupae; average collections of: (E) 4th instar larvae from basins in 2020, and (F) pupae from basins in 2020. Colors represent the Town; bars represent the GLMM predictions; solid lines represent the 95% confidence interval of the prediction. Columns represent the number of weeks since the larvicide VectoLex FG (active ingredient *Lysinibacillus sphaericus*) was applied in catch basins in each town.

We conducted 1262 basin sampling events: water was present in 97% of all sampling events, and the average prevalence of larvae and pupae in sampled catch basins was 68% and 41%, respectively. Larval and pupal prevalence in catch basins varied by week (Fig. 2), and each metric was consistently lower in Milford than Stratford (Table 2). These differences remained significant after accounting for average weekly precipitation and repeated measures in the GLMM framework (Tables S2 and S3). GLMMs of larval prevalence among catch basins predicted declines in prevalence in each town during the first week post‐treatment with *L. sphaericus*, and the overall effect size of Town and Treatment period was greater in Milford than Stratford [Fig 3A]. We also detected an overall lower prevalence of pupae among catch basins in Milford [Table 2, Fig 3B]; however, the Treatment variable was not an informative predictor (Table S3). Due to a CAES *C. pipiens* colony collapse in 2019, we could not perform the mortality bioassays in a manner consistent with our methods and appropriate for analysis.

**Table 2:** Catch basin sampling summaries in Milford and Stratford, Connecticut 2019–2020

Town (total basins: density, km^2^)	Location	2019			2020						
		Total Samples (basins sampled weekly)	Prevalence any larval instar	Prevalence pupae	Basins within 500 m buffer (density, per km^2^)	Total samples (basins sampled weekly)	Prevalence 4th instar larvae	Total 4th instar larvae (average per sample)	Prevalence pupae	Total pupae (average per sample)	Total samples screen for mortality (average mortality)
Milford (8.054: 123.6)	Liberty Rock	165 (8)	63%	32%	193 (245.9)	353 (20)	30.1%	439 (1.24)	13.2%	167 (0.47)	220 (25.0%)
	Margaret Egan	NS	NS	NS	174 (221.7)	329 (18)	18.9%	718 (2.18)	8.7%	708 (2.15)	186 (32.5%)
	Meadowside	164 (10)	64%	42%	111 (141.4)	194 (11)	38.2%	810 (4.18)	30.6%	450 (2.32)	106 (29.3%)
	Downtown Milford (2019) Parsons Complex (2020)	184 (9)	61%	35%	208 (265.0)	338 (18)	34.5%	2299 (6.80)	26.7%	1247 (3.69)	174 (28.2%)
	Pond Point	135 (8)	27%	21%	119 (151.6)	223 (12)	26.2%	705 (3.16)	28.0%	580 (2.6)	134 (44.6%)
	Washington Field	NS	NS	NS	131 (166.9)	244 (13)	16.9%	353 (1.45)	9.1%	165 (0.68)	124 (20.2%)
	**Total**	**648 (34)**	**55%**	**44%**	**936 (198.7)**	**1681 (92)**	**24.4%**	**5324 (3.17)**	**18.3%**	**3317 (1.97)**	**944 (29.9%)**
Stratford (5072: 98.6)	Cloverfield	NS	NS	NS	98 (124.8)	183 (12)	21.0%	760 (4.15)	8.0%	76 (0.42)	106 (3.4%)
	High Park	202 (10)	81%	44%	155 (197.5)	280 (16)	46.6%	2383 (8.51)	39.2%	1143 (4.08)	114 (12.7%)
	Historic District	110 (7)	90%	68%	129 (164.3)	231 (12)	66.7%	2376 (10.3)	50.7%	1239 (5.36)	77 (14.0%)
	Long Brook	NS	NS	NS	108 (137.6)	169 (11)	30.1%	1052 (6.22)	21.2%	376 (2.22)	72 (6.4%)
	Stony Brook	114 (6)	83%	57%	136 (173.2)	246 (14)	41.2%	1580 (6.42)	31.9%	893 (3.63)	117 (13.2%)
	Woodend	188 (9)	77%	42%	152 (193.6)	289 (15)	59.2%	2657 (9.19)	45.6%	1093 (3.78)	114 (26.1%)
	**Total**	**614 (31)**	**82%**	**50%**	**778 (165.2)**	**1398**	**46.4%**	**10 808 (7.73)**	**34.9%**	**4820 (3.45)**	**597 (13.4%)**

NS, not sampled.

**Figure 4:**
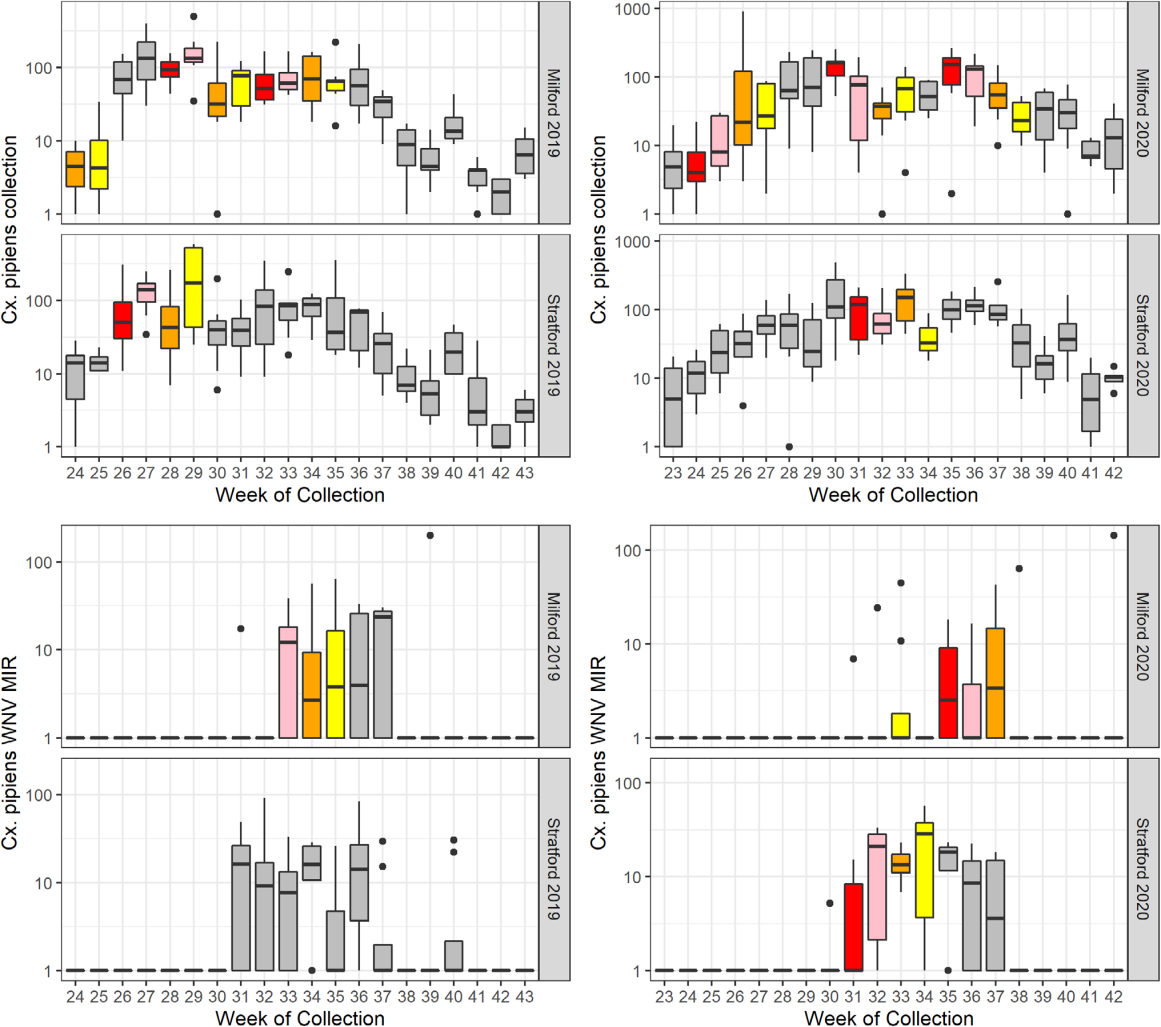
Average weekly *Culex pipiens* adult collections and West Nile virus minimum infection rates from gravid traps in Milford and Stratford, CT 2019 in 2019 and 2020. Colors indicate the larvicide treatment variable designation (red, week 1; pink, week 2; orange, week 3; yellow, week 4; grey, no or assumed decline of treatment effectiveness).

We conducted 308 and 289 trap‐night collections with gravid and light traps, respectively, and we collected 36 140 and 43 036 individuals in each trap type, respectively. *Culex pipiens, Culex restuans* and *Culex* spp. (*pipiens/restuans* individuals that could only be reliably identified to genus as a consequence of specimen damage) were the primary species collected in gravid traps, and there was no difference in *C. pipiens* gravid trap collections between Milford and Stratford (Fig. 4; Table S4); *C. pipiens* gravid trap collections were also not associated with the catch basin treatment periods (Table S4). We did find that the number of basins positive for pupae within the 500 m sampling buffer improved the AIC in our GLMM of *C. pipiens* gravid trap collections; however, the direction of the relationship was negative and not significant (*P* > 0.1) (Table S5). We did detect fewer *C. pipiens* collections in light traps in Milford compared to Stratford (Tables S5 and S6), and this difference in light trap collections extended to comparisons of all species collected (Table 3). Similar to our analyses of gravid traps, metrics of larval and pupal prevalence among catch basins were not informative predictors of *C*. *pipiens* light trap collections (Table S6).

**Table 3:** Gravid and CO_2_‐baited, ground‐level light trap collections in Milford and Stratford, CT 2019–2020

Species	2019								2020							
	Trap type								Trap type							
	Milford				Stratford				Milford				Stratford			
	Gravid		Light		Gravid		Light		Gravid		Light		Gravid		Light	
	Total Collection	WNV	Total Collection	WNV	Total Collection	WNV	Total Collection	WNV	Total Collection	WNV	Total Collection	WNV	Total Collection	WNV	Total Collection	WNV
*Aedes absurattus*			1	0												
*Aedes albopictus*	148	1	167	0	401	2	1155	0	201	0	310	0	501	0	1860	0
*Aedes canadensis*	7	0	369	0			7	0			374	0			2	0
*Aedes cantator*	4	0	336	0	9	0	406	0			130	0	4	0	247	0
*Aedes cinereus*	1	0	18	0			109	0			3	0	8	0	641	0
*Aedes excrucians*			13	0			4	0			4	0			1	0
*Aedes fitchii*											1	0				
*Aedes grossbecki*	1	0									3	0				
*Aedes japonicus*	1027	0	510	0	290	2	1978	0	497	0	197	0	203	0	426	0
*Aedes sollicitans*	14	0	629	0	41	0	863	0	3	0	205	0			106	0
*Aedes spp*.											11	0			39	0
*Aedes sticticus*			2	0												
*Aedes stimulans*	6	0	6	0			10	0	1	0	7	0			6	0
*Aedes taeniorhynchus*	2	0	730	0			11	0	3	0	1167	0	10	0	2131	0
*Aedes thibaulti*			4	0			1	0	2	0	1	0			40	0
*Aedes triseriatus*	37	0	73	0	21	0	121	0	13	0	23	0	3	0	45	0
*Aedes trivittatus*			1327	0	1	0	51	0	5	0	1795	0			109	0
*Aedes vexans*	16	0	4276	1	10	0	7472	0	4	0	1424	0			2404	
*Anopheles crucians*	1	0	9	0	1	0	15	0								
*Anopheles punctipennis*	2	0	111	0	2	0	105	0	1	0	48	0	1	0	29	0
*Anopheles quadrimaculatus*	9	0	156	0			716	0	2	0	63	0	5	0	128	0
*Anopheles spp*.			1	0			1	0								
*Anopheles walkeri*			1	0			2	0								
*Coquilletedia perturbans*	12	0	1063	0			345	1			520	0			92	0
*Culiseta melanura*			12	0			2	0								
*Culiseta minnesotae*			4	0			1	0								
*Culex erraticus*	2	0	24	0			5	0			29	0			6	0
*Culex pipiens*	8402	31	387	1	9446	39	1738	6	9988	21	140	0	10 038	58	2959	4
*Culex restuans*	5100	2	419	0	2029	0	668	2	2119	1	105	0	1184	1	278	0
*Culex salinarius*	189	1	3527	2	238	4	8579	4	91	0	630	0	118	0	1905	0
*Culex spp*.	5064	3	122	1	3567	10	222	0	2310	4	62	0	1944	1	413	0
*Culex territans*	3	0	8	0	7	0	5	0	5	0			4	0		
*Orthopodemia signifera*	1	0	2	0			1	0	1	0						
*Psorophora columbiae*			5	0							3	0			3	0
*Psorophora ferox*	1	0	187	1	2	0	150	0	4	0	408	0	9	0	228	0
*Psorophora howardii*	7	0					5	0			2	0			5	0
*Uranotenia sapphirina*	1	0	39	0	6	0	23	0	1	0	3	0			7	0
**TOTAL**	**20 057**	**38**	**14 538**	**6**	**16 071**	**57**	**24 771**	**13**	**15 251**	**26**	**7668**	**0**	**14 032**	**60**	**14 110**	**4**

A total of 113 WNV positive isolates were detected in 2019: 44 in Milford and 69 in Stratford. *Culex pipiens* accounted for 68% of all WNV isolates, whereas *Culex* spp., *Culex salinarius* and *C. restuans* accounted for 12%, 8.8% and 3.5%, respectively. We also detected three WNV isolates in *Aedes albopictus* (one Milford; one Stratford), two isolates in *Aedes japonicus* in Stratford, and one isolate in *Aedes vexans* in Milford. Despite the greater number of WNV isolates in Stratford compared to Milford, this difference was not significant when restricting analyses to *C. pipiens* gravid trap collections and accounting for repeated spatial and temporal measures (Fig. 4; Tables S4 and S5). We did find an association between the larvicide treatment variable and WNV isolates and WNV MIRs (Table S4); however, this was likely to have been a spurious association because the majority of WNV isolates detected in each town were collected during what were considered ‘No Treatment’ periods near the end of summer and the dearth of larvicide treatment periods in Stratford (Fig. 4).

### 3.2 Field season 2020

**Figure 5:**
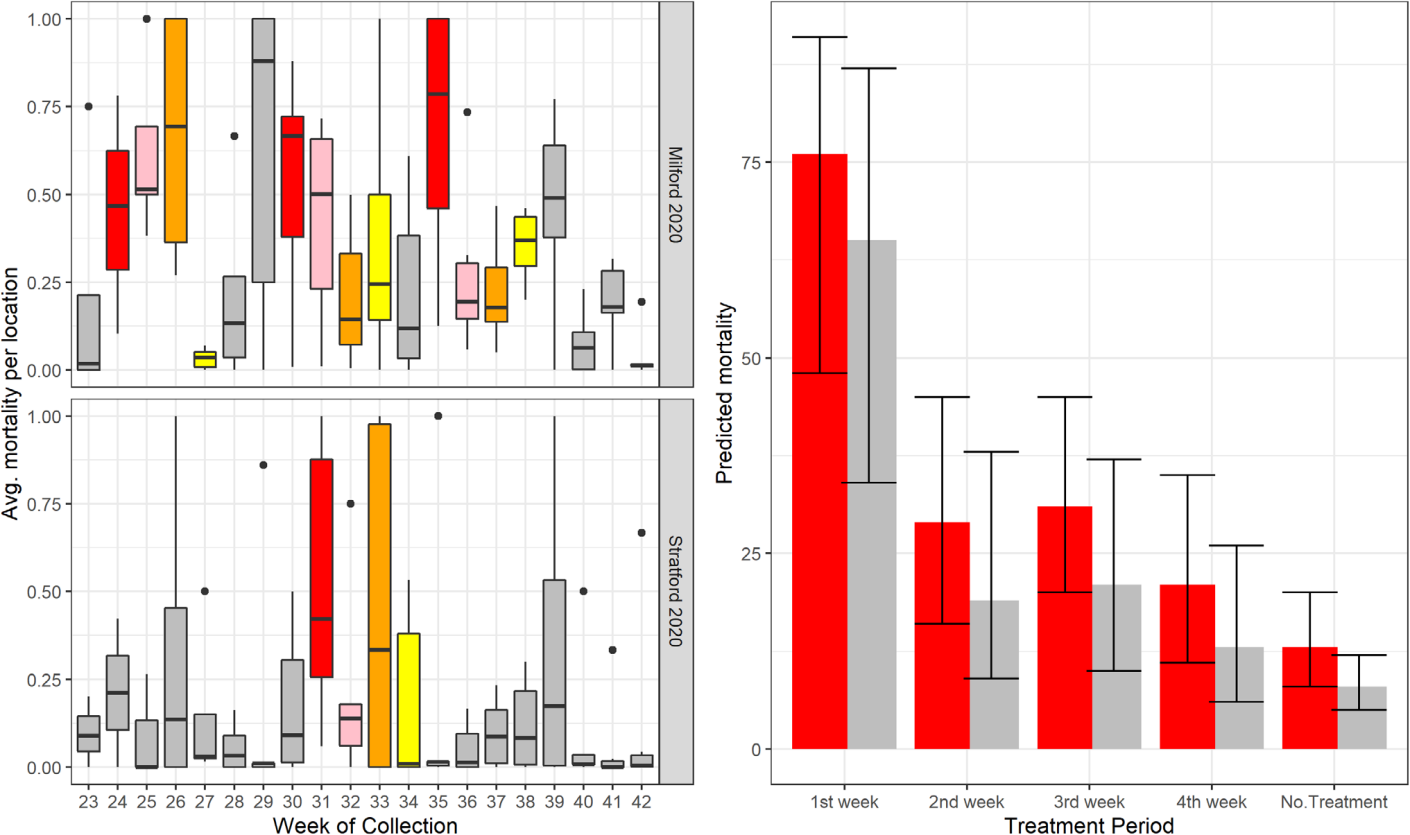
Observed (boxplots) and predicted (bar plots) larval mortality in bioassays of catch basin water samples from Milford and Stratford in 2020. Boxplot colors indicate the larvicide treatment variable designation (red, week 1; pink, week 2; orange, week 3; yellow, week 4; grey, no or assumed decline of treatment effectiveness). Bar plot colors represent the GLMM prediction for each treatment designation by Town (red, Milford; grey, Stratford). Lines represent the 95% confidence interval of the GLMM prediction.

We conducted 3079 basin sampling events: water was present in 84% of sampling events, and the average prevalence of 4th instar larvae and pupae in sampled catch basins was 34.4% and 25.9%, respectively. Larval/pupal prevalence and abundance were consistently lower in Milford compared to Stratford (Table 2; Fig. 2), results that remained significant after accounting for average weekly precipitation and repeated measures in the GLMM framework (Tables S8 and S9). Predicted declines in 4th instar larval and pupal prevalence and abundance were observed only in Milford and occurred during the first two‐week post‐larvicide applications [Fig. 3(C)–(F)]. When assessing site‐level random effects, GLMMs of 4th instar and pupal collections in catch basins revealed a high degree of variability in collections between basins, and ≤40% of sampled basins in each town displayed significant changes on GLMM intercepts (Table S10); ≈25% significantly increased whereas ≈20% significantly decreased GLMM intercepts. We were able to successfully screen water samples for mortality utilizing our re‐established *C. pipiens* colony in 2020, and mortality was highest in Milford compared to Stratford (Table 2; Fig. 5). Mortality estimates were also highest the first week post‐larvicide treatments, and mortality estimates declined in each week postapplication (Fig. 5). We did not detect an interaction between Town and the larvicide treatment variable in our bioassay GLMM (Table S11), suggesting that operationally, the larvicide products behaved similarly in each town; the greater number of treatments in Milford is what likely influenced the overall difference in mortality estimates between the two towns.

We conducted 291 and 276 trap‐night collections with gravid and light traps, respectively, and collected 29 286 and 21 785 individuals in each trap type, respectively. *Culex pipiens, C. restuans* and *Culex* spp. were again the dominant species collected in gravid traps, and there was no difference in *C. pipiens* gravid trap collections between Milford and Stratford (Tables 3 and S12; Fig. 4). As in 2019, we collected fewer individuals overall in light traps in Milford compared to Stratford (Tables 3 and S13). We were able to detect associations between larval as well as pupal metrics in catch basins and *C. pipiens* collections in gravid and light traps; however, all of these associations were of small effect size (Tables S12 and S13, <Δ2% in GLMM prediction compared to intercept). Total pupal collections in catch basins within 500 m of a trap site were positively associated with *C. pipiens* gravid trap collections and the number of basins containing 4th instar larvae were positively associated with *C. pipiens* collections in light traps (Table S12). The number of catch basins containing 4th instar larvae also were negatively associated with *C. pipiens* collections in gravid traps (Table S12). The larvicide treatment variable also was an informative predictor of *C. pipiens* collections in light traps with collections sharing a negative and significant association with the fourth week post‐treatment (Table S13).

A total of 90 WNV positive isolates were detected in 2020: 26 in Milford and 64 in Stratford. *Culex pipiens* samples accounted for 92% of all WNV isolates with the remaining samples identified as either *Culex* spp. (*n* = 5) or *C. restuans* (*n* = 2). *Aedes albopictus* and *A. japonicus* samples were not screened for WNV in 2020. Unlike in 2019, the number of WNV‐positive samples and associated WNV MIRS in *C. pipiens* gravid trap collections were considered statistically different in our GLMMs (Fig. 4; Table S12); however, there were no associations between WNV infection prevalence, larval and pupal occupancy metrics in catch basins, nor the larvicide treatment variable (Table S12).

## 4 DISCUSSION

We conducted WNV surveillance and larvicide effectiveness evaluations in two towns in CT that employ different mosquito larval control strategies to examine the associations between larval control and WNV activity. Evidence of WNV activity was prevalent in each town despite one utilizing a comprehensive larval control program that treated basin and nonbasin habitats, and the other performing a single, seasonal treatment in catch basins only. In each town, *C. pipiens* larvae and pupae collections in catch basins were reduced for up to two weeks following treatments with the *L. sphaericus* product VectoLex FG, supporting previous research showing declining effectiveness of biopesticides in catch basins over one to two weeks.^43^, ^51^ We found that neither the abundance of pupae in catch basins, nor periods of catch basin treatments with larvicides were associated with *C. pipiens* adult collections and WNV mosquito infection prevalence in gravid traps. However, we determined that town of collection shared an association with the number of *C. pipiens* females captured in CO_2_‐baited light traps. Light trap collections were lower in Milford which treated basin and nonbasin habitats, which suggests that Milfordʼs efforts possibly reduced populations of younger, host‐seeking *C. pipiens* individuals. Control of mosquito populations in catch basins and nonbasin habitats is considered an integral component of any IVM program for WNV; however, further research is necessary to link productivity and control of larvae in these environments to entomological risk metrics of WNV.

The state of CT manages mosquito populations on state property only, and both the methods and habitats managed by the State are not applicable to the methods and habitats most relevant to reducing risk of WNV.^29^ Thus, WNV mosquito control in CT is conducted primarily at the level of independent municipal towns, and towns that do maintain seasonal mosquito control programs commonly apply larvicides in catch basins as their primary means of controlling WNV; other larval control approaches often are aimed at reducing the prevalence of nuisance species, and these strategies concentrate in coastal regions across CT. This approach for WNV control in CT is consistent with results from surveys of local and county public health departments in the US showing that the majority of local to regional authorities maintain some form of mosquito larval control program.^6^ Epi‐evaluations of larval control programs are rare, although a previous field experiment of larval control in catch basins was unable to associate reductions in larvae and pupae with reductions in WNV entomological risk metrics at small spatial scales.^28^ Our current study extends this previous experiment to a larger spatial scale (with a sampling trade‐off of fewer traps per km^2^) and more directly observes the practices of local mosquito control stake holders. Our results indicate that one to three seasonal applications of larvicides in catch basins, coupled with nonbasin treatments of flood‐prone habitats, at the scale investigated (55–65 km^2^) are insufficient to suppress *C. pipiens* adult populations and subsequent risk of WNV spillover. More research is needed to understand if and under what application conditions control in catch basin and other larval sources can control WNV in urban environments.

Larvicides are effective tools for reducing larval and pupal densities in the larval environment.^19^, ^22^, ^43^, ^52^ Operational evaluations of treatment programs have, however, identified numerous factors that reduce the effectiveness of the treatments.^15^, ^21^, ^24^, ^25^, ^53^, ^54^ *Culex pipiens* larvae and pupae also are commonly found in noncatch basin habitats,^14^, ^55^, ^56^ yet there are few studies that have quantified the proportion of *C. pipiens* adults in a population generated from these nonbasin sources. Improving the operational success of larval control programs, such as minimizing the number of untreated basins, treating nonbasin habitats and maximizing product residual effectiveness, could improve the overall impact on *C. pipiens* populations. Our results also show that larvicide effectiveness in basins waned within two weeks of application; revised application strategies in both towns which treat basins more frequently or utilize a higher dose or stronger time‐release product may extend effectiveness. Furthermore, larvicide treatments in each town occurred during time periods when WNV transmission was active; because any impact of larval control on adult populations would occur at a lag of one to two weeks, focusing treatments earlier in a season may improve population impacts.^57^ Finally, our analyses of GLMM random effects revealed a high level of variation in catch basin collections with ≥25% of catch basins displaying collections far above the modelʼs intercept (i.e. average); although it is likely a costly and time‐consuming process, identifying productive larval habitats and ensuring maximum larvicide effectiveness in these areas may prove more impactful than spatially indiscriminate and uniform treatments.^24^, ^25^

Given the knowledge gap pertaining to epi‐impacts of larval control on WNV activity, it is uncertain how operational improvements in larval control treatments will lead to epidemiological improvements. Proactive larval control is considered only the first line of control for WNV with varying increases in efforts (such as adulticide spraying) pending transmission intensity.^5^ In reality, very few municipalities are equipped to implement control measures such as broad‐scale aerial and/or ground‐level applications of insecticides, and for catch basins, timely annual maintenance of wastewater infrastructure at levels sufficient to reduce the prevalence of larval habitats. A recent review also determined that control measures for WNV are rarely based on actionable surveillance thresholds as recommended by CDC^26^; given cost trade‐offs between surveillance and control, and variation in public support for either program, it is likely that local municipalities are incapable of supporting an effective and impactful IVM strategy for WNV without greater financial and public support from local, state and federal entities.^58^ Further research also is needed to define the spatial coverage and frequency of larvicide applications needed to control *C. pipiens* larval populations to have an effect on WNV risk. In our study, we found no difference in gravid trap collections and WNV infection prevalence between Milford and Stratford, despite an overall difference in larval/pupal abundance and larval control strategies. Retrospective analyses have identified broad‐scale influences of climate on regional WNV epidemics^59^ whereas viral dynamics studies of WNV show that genetic variants of WNV are capable of dispersing across wide distances.^60^, ^61^ Within CT specifically, multiple cities are considered high‐risk sites for WNV; in situations in which control efforts are unequal between cities, each high‐risk site could act as a source of WNV‐infected birds and mosquitoes for another.^29^ Either coordinating larval control efforts between cities and/or scaling up control programs to a regional level may be a worthwhile endeavor in CT, and more impactful than variable and inconsistent efforts among towns.

A limitation of this work is that it was designed to evaluate larval control in catch basins as implemented by local public health departments in CT. Future studies should coordinate more appropriately between control applicators, public health and other community stakeholders, and vector surveillance programs to maximize the impact of larval control in catch basins as well as nonbasin habitats. Previous studies have investigated re‐treatment thresholds on both per‐basin^18^ and per‐area^36^ bases, and follow‐up studies and treatment programs in CT and other regions could enact such thresholds to improve operational outcomes of larval control. Future research should also expand beyond the spatial coverage investigated in this study. Our power analyses based on our 2019 results indicated that owing to the estimated variance in collections among traps, many more surveillance sites would be needed to detect a 20% difference in *C. pipiens* trap collections than we could employ based on present people‐power. Region‐wide, coordinated and multi‐institutional studies that include multiple sampling clusters in numerous locations would likely be a more effective observational approach to investigating larval control impacts on WNV. More direct measures of adult production from catch basins, such as aspirating recently emerged or resting mosquitoes from the interior of basins^28^ or the use of emergence traps,^62^ also should be considered in follow‐up studies of the impact of larval control interventions on adult *C. pipiens* populations. Coupled with the expansion of spatial replicates and refined sampling methodologies, future site selection also should be randomized. The surveillance sites in this study are subject to selection bias as we were limited to sampling in public spaces, and our present analyses do not explicitly account for variability in socio‐economics and other land cover variables which are important landscape predictors of WNV risk.^63^, ^64^, ^65^ Finally, other metrics of WNV transmission should be considered in future studies. For instance, seroconversion rates in sentinel animals or incidence rates in nucleic‐acid preservation cards may provide a more direct measure of WNV transmission at a particular site^66^, ^67^, ^68^, ^69^, ^70^ given that *C. pipiens* mosquitoes are capable of dispersal distances greater than the 500 m buffer utilized in this report.^40^

## 5 CONCLUSION

*Culex* spp. larval source reduction using insecticides is one important approach to WNV prevention in the United States. Evaluations of larval control programs tend to focus on operational outcomes such as product residual effectiveness and improved spatiotemporal coverage, and rarely address the entomological and epidemiological impact of control. Through the use of targeted WNV surveillance and larvicidal evaluations in catch basins, we demonstrated that seasonal larvicide treatments in catch basins and nonbasin habitats at small municipal levels (i.e. towns <65 km^2^) in CT, USA have limited impact on adult entomological risk metrics associated with WNV activity. Although more research is needed to define the community‐wide impacts of larval control on WNV transmission, our results suggest that much greater mosquito control efforts are needed at local and regional scales to reduce entomological indices associated with WNV risk.

## CONFLICT OF INTEREST

None.

## Supporting information


**Appendix S1**. Supporting Information

**Appendix S2**. Supporting Information

**Appendix S3**. Supporting Information

## Data Availability

All data necessary to produce the reported results are available using the following link: doi:10.5061/dryad.4xgxd259m
