# Supplementary material for: The community‐wide effectiveness of municipal larval control programs for West Nile virus risk reduction in Co nnecticut, USA
Source: Pest Manag Sci. 2021 Aug 5;77(11):5186–201. doi: 10.1002/ps.6559 (PMC9291174; doi:10.1002/ps.6559)
Supplement: Supplementary file 1 — Appendix S1. Supporting Information [file PS-77-5186-s003.docx]

**Supporting information for:**

**Title.** The community-wide effectiveness of municipal larval control programs for West Nile virus risk reduction in Connecticut, United States

**Authors.** Joseph R. McMillan^1,2^, Christina A. Harden^3^, James C. Burtis^2,4^, Mallery I. Breban^5^, John J. Shepard^1^, Tanya A. Petruff^1^, Michael J. Misencik^1^, Angela B. Bransfield^1^, Joseph D. Poggi^2,5^, Laura C. Harrington^2,5^, Theodore G. Andreadis^1,2^, and Philip M. Armstrong^1,2^

**Author Affiliations.**

1. The Connecticut Agricultural Experiment Station, New Haven, CT
2. The Northeast Regional Center of Excellence in Vector-borne Diseases
3. Pennsylvania State University, State College, PA
4. Division of Vector-borne Diseases, Centers for Disease Control and Prevention, Fort Collins, CO
5. Cornell University, Ithaca NY
6. Yale University, New Haven, CT

**Corresponding Author.**

Joseph R. McMillan

joseph.mcmillan@ct.gov

123 Huntington Street

New Haven, Connecticut 06511


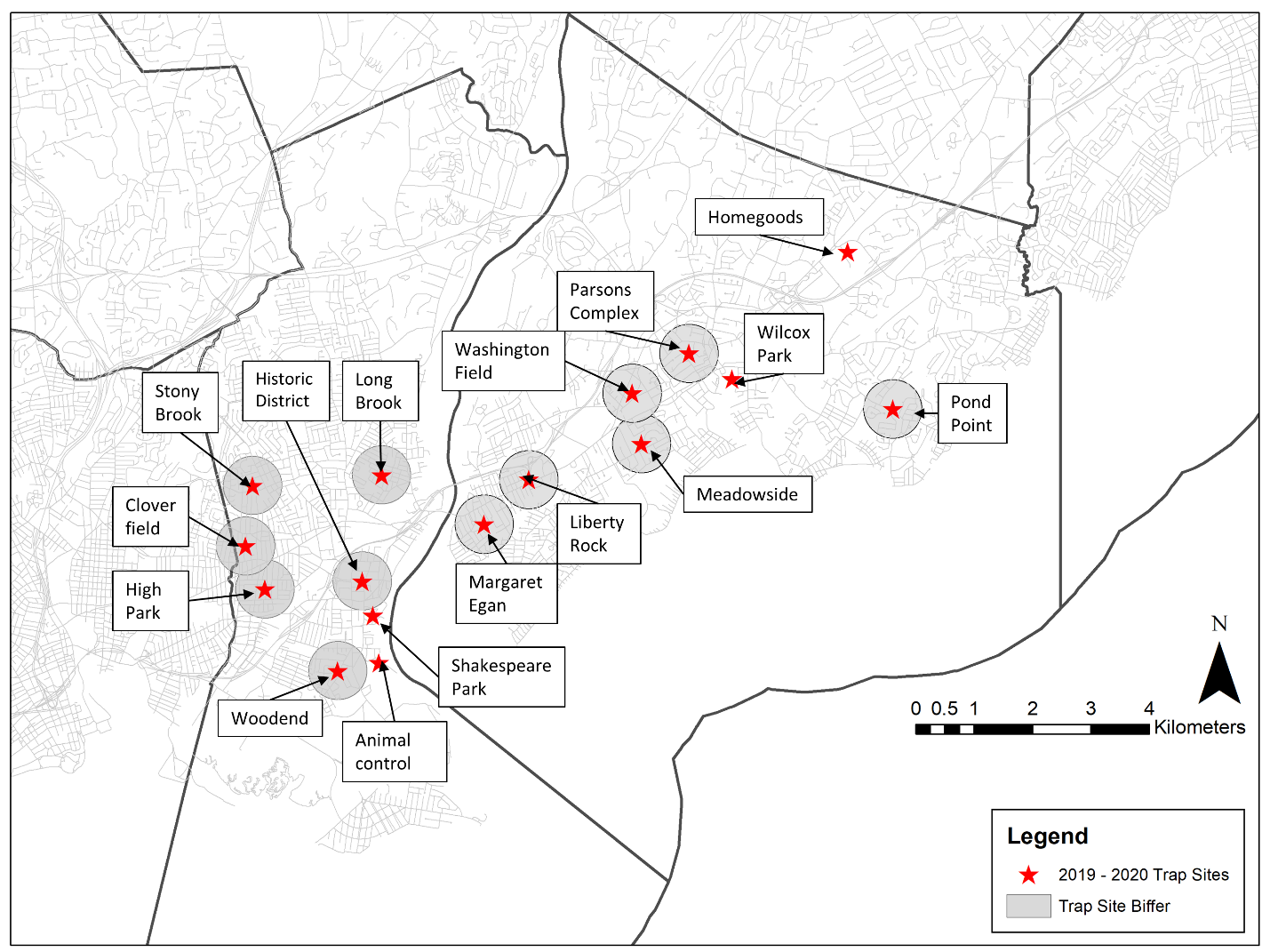


**S. Figure 1.** Map of labeled surveillance sites in Milford and Stratford, CT 2019 – 2020. Red stars indicate adult mosquito and West Nile virus surveillance sites, dark grey background demarcate the 500m buffer surrounding trap sites with accompanying catch basins surveillance in 2020, solid black lines demarcate each town’s political boundary, and thin grey lines demarcate roads. Names of each surveillance site are listed and arrows point to the corresponding site. Red stars without an accompanying grey buffer indicate that these sites do not have any accompanying catch basin information from the 2019 or 2020 season.


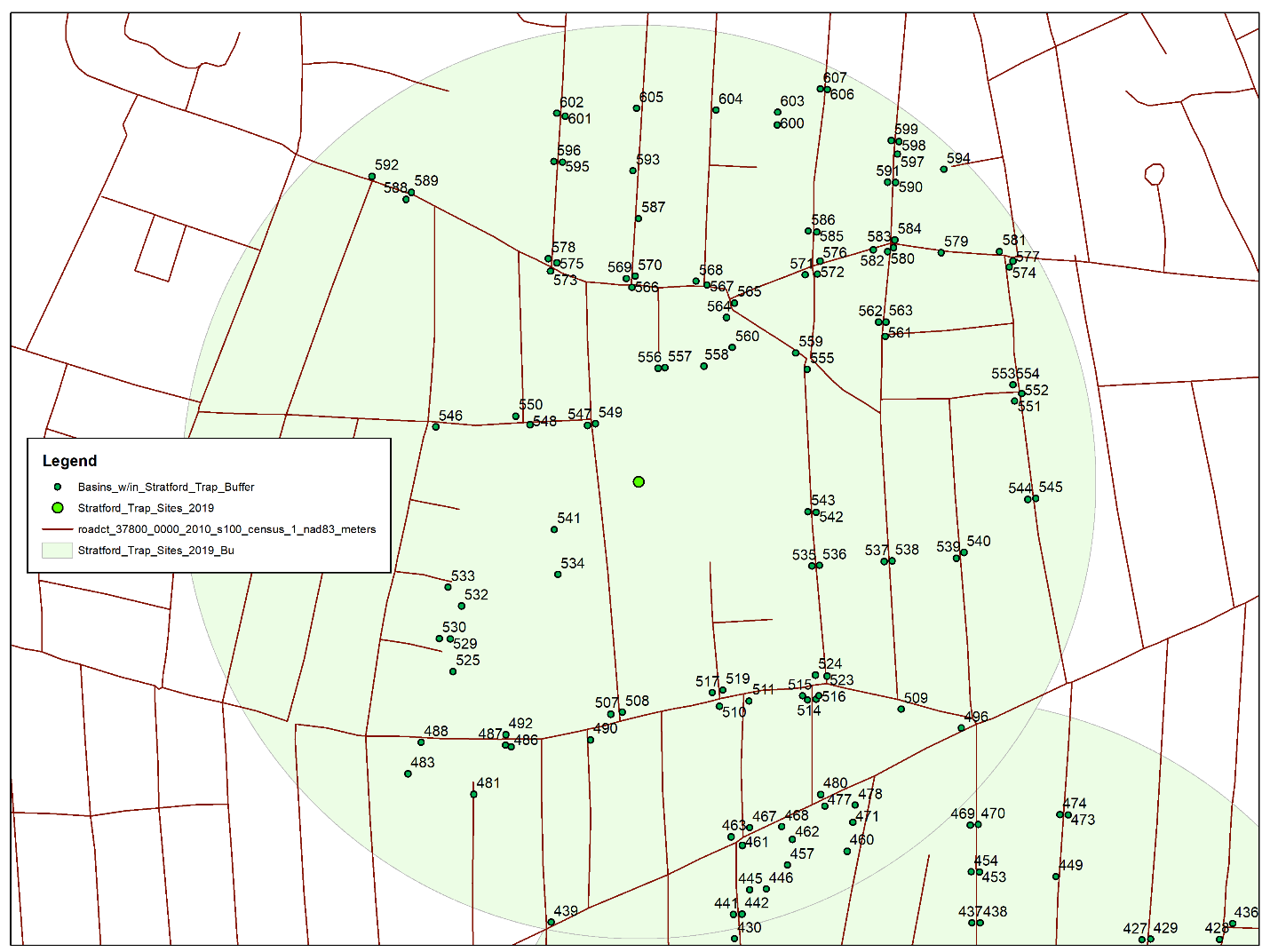


**S. Figure 2**. “Cloverfield” surveillance site in Stratford, Connecticut. The central bight green circle designates the placement of a single gravid and CO_2_-baited light trap for adult mosquito and West Nile virus surveillance. The light green shaded regions demarcates the 500m buffer surrounding the surveillance location. The small green points with labeled numbers designate the location of catch basins within the 500m buffer surrounding the surveillance location. Thin, brown lines designate roads. A portion of the “Cloverfield” 500m buffer overlaps with the “High Park” 500m buffer. Catch basins sampled in 2020 included: 486, 514, 517, 519, 523, 524, 535, 546, 565, 576, 578, 580, 583, and 585


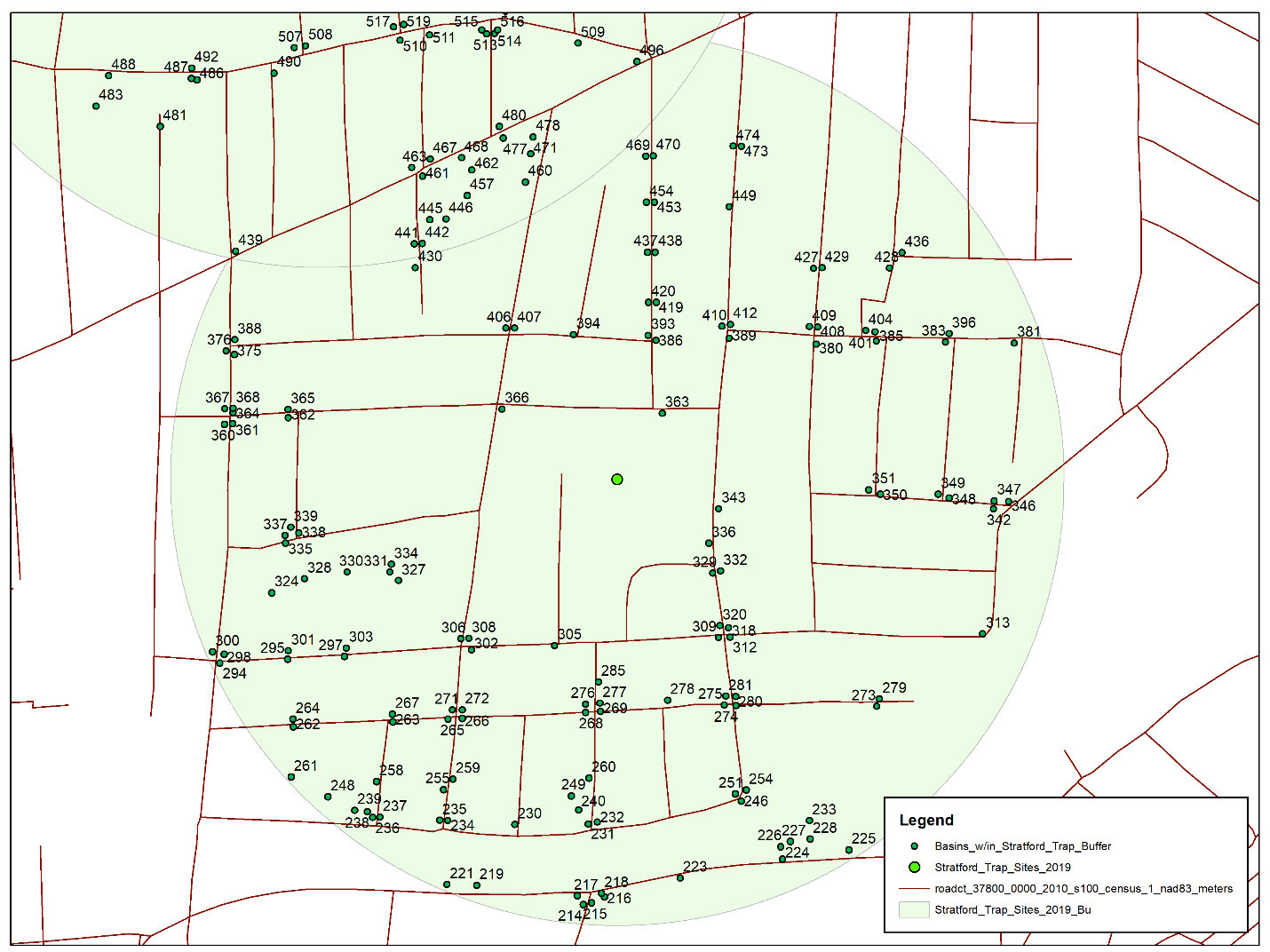


**S. Figure 3**. “High Park” surveillance site in Stratford, Connecticut. The central bight green circle designates the placement of a single gravid and CO_2_-baited light trap for adult mosquito and West Nile virus surveillance. The light green shaded regions demarcates the 500m buffer surrounding the surveillance location. The small green points with labeled numbers designate the location of catch basins within the 500m buffer surrounding the surveillance location. Thin, brown lines designate roads. A portion of the “High Park” 500m buffer overlaps with the “Cloverfield” 500m buffer. Catch basins sampled in 2019 included: 305, 306, 308, 363, 366, 406, 460, 469, and two basins not listed in the GIS file (both near 366 and 406). Catch basins sampled in 2020 included: 231, 246, 254, 273, 305, 312, 318, 346, 380, 381, 383, 385, 406, 412, 437, and 439


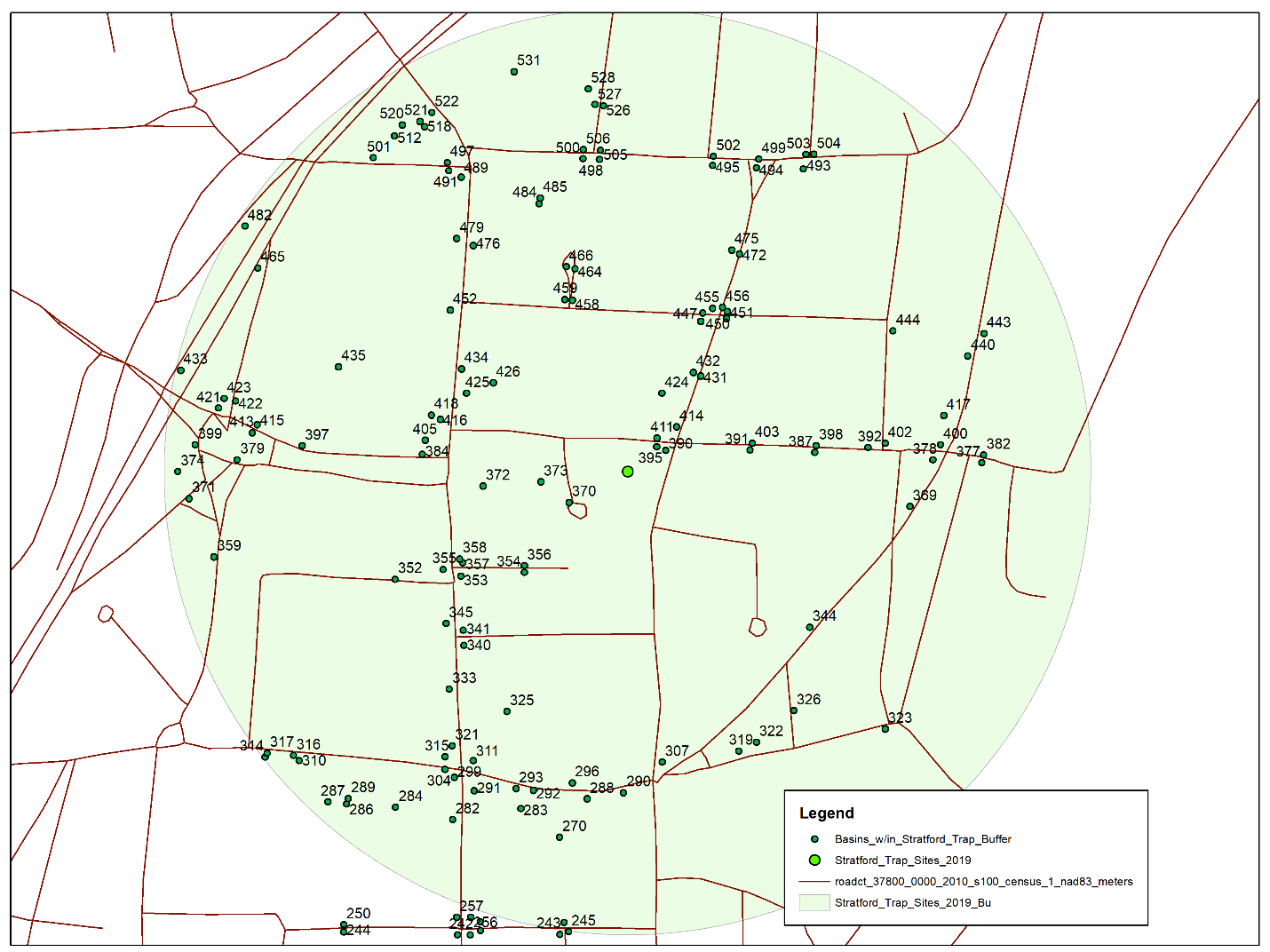


**S. Figure 4**. “Historic District” surveillance site in Stratford, Connecticut. The central bight green circle designates the placement of a single gravid and CO_2_-baited light trap for adult mosquito and West Nile virus surveillance. The light green shaded regions demarcates the 500m buffer surrounding the surveillance location. The small green points with labeled numbers designate the location of catch basins within the 500m buffer surrounding the surveillance location. Thin, brown lines designate roads. Catch basins sampled in 2019 included: 451, 455, 459, 464, and four basins not listed in the GIS file (all between 458 and 455). Catch basins sampled in 2020 included: 282, 293, 307, 314, 326, 341, 387, 391, 450, 451, 458, 466, and 527.


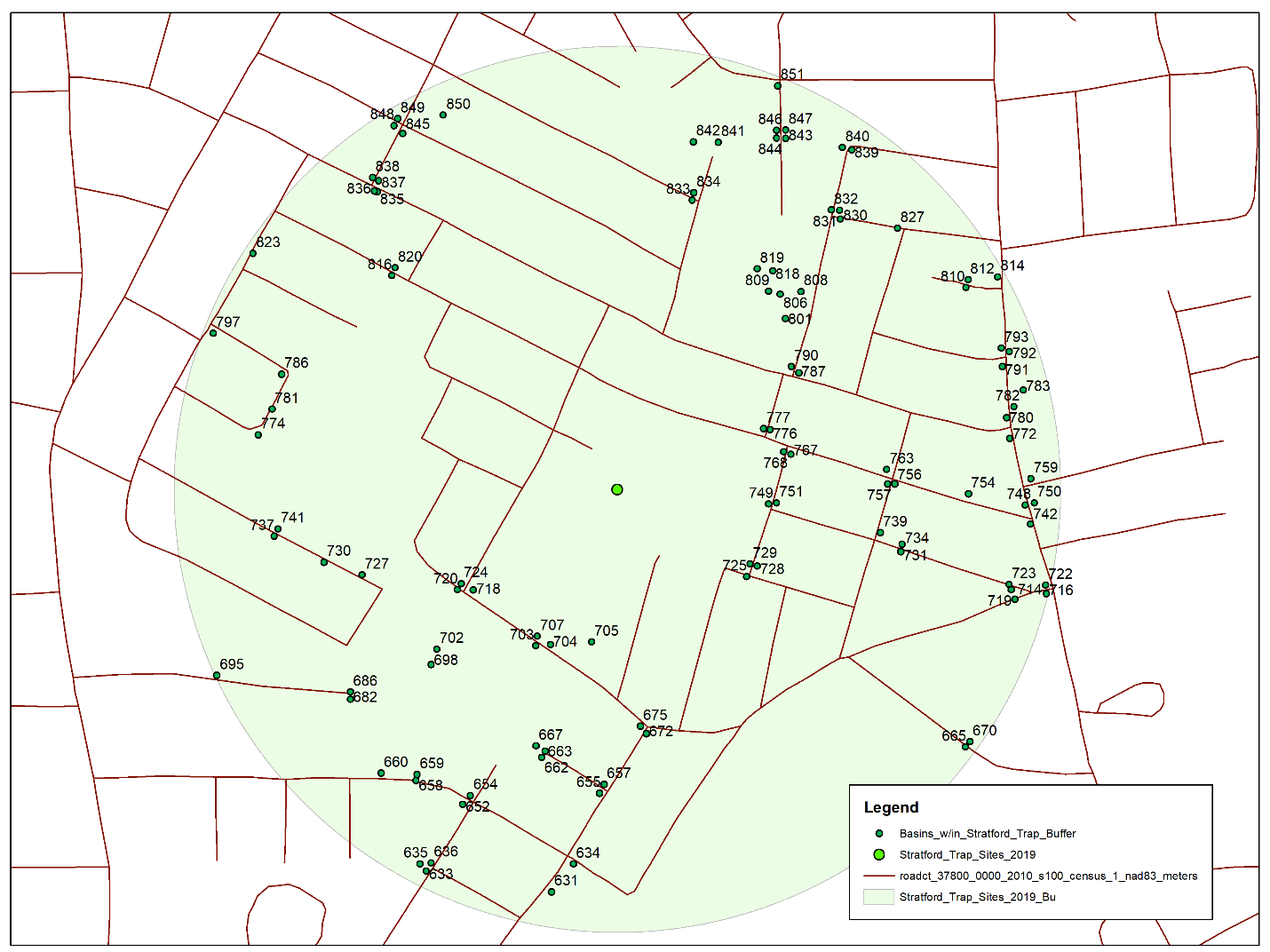


**S. Figure 5**. “Longbrook” surveillance site in Stratford, Connecticut. The central bight green circle designates the placement of a single gravid and CO_2_-baited light trap for adult mosquito and West Nile virus surveillance. The light green shaded regions demarcates the 500m buffer surrounding the surveillance location. The small green points with labeled numbers designate the location of catch basins within the 500m buffer surrounding the surveillance location. Thin, brown lines designate roads. Catch basins sampled in 2020 included: 657, 660, 662, 665, 703, 714, 722, 723, 741, 767, 781, and 827.


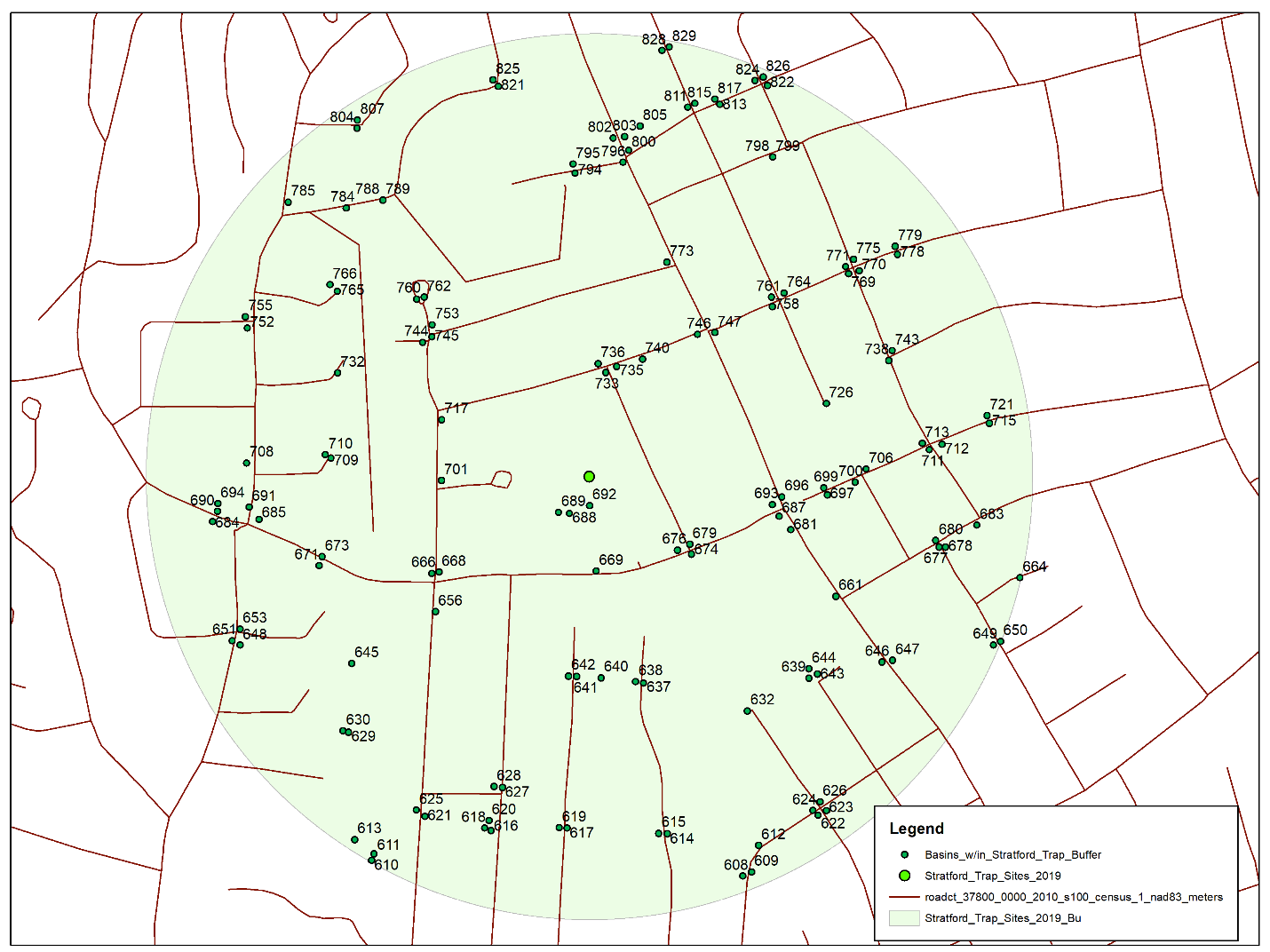


**S. Figure 6**. “Stony Brook” surveillance site in Stratford, Connecticut. The central bight green circle designates the placement of a single gravid and CO_2_-baited light trap for adult mosquito and West Nile virus surveillance. The light green shaded regions demarcates the 500m buffer surrounding the surveillance location. The small green points with labeled numbers designate the location of catch basins within the 500m buffer surrounding the surveillance location. Thin, brown lines designate roads. Catch basins sampled in 2019 included: 656, 668, 701, 733, 735, and 744. Catch basins sampled in 2020 included: 609, 919, 621, 625, 638, 643, 673, 693, 773, 799, 807, 811, 825, and 829.


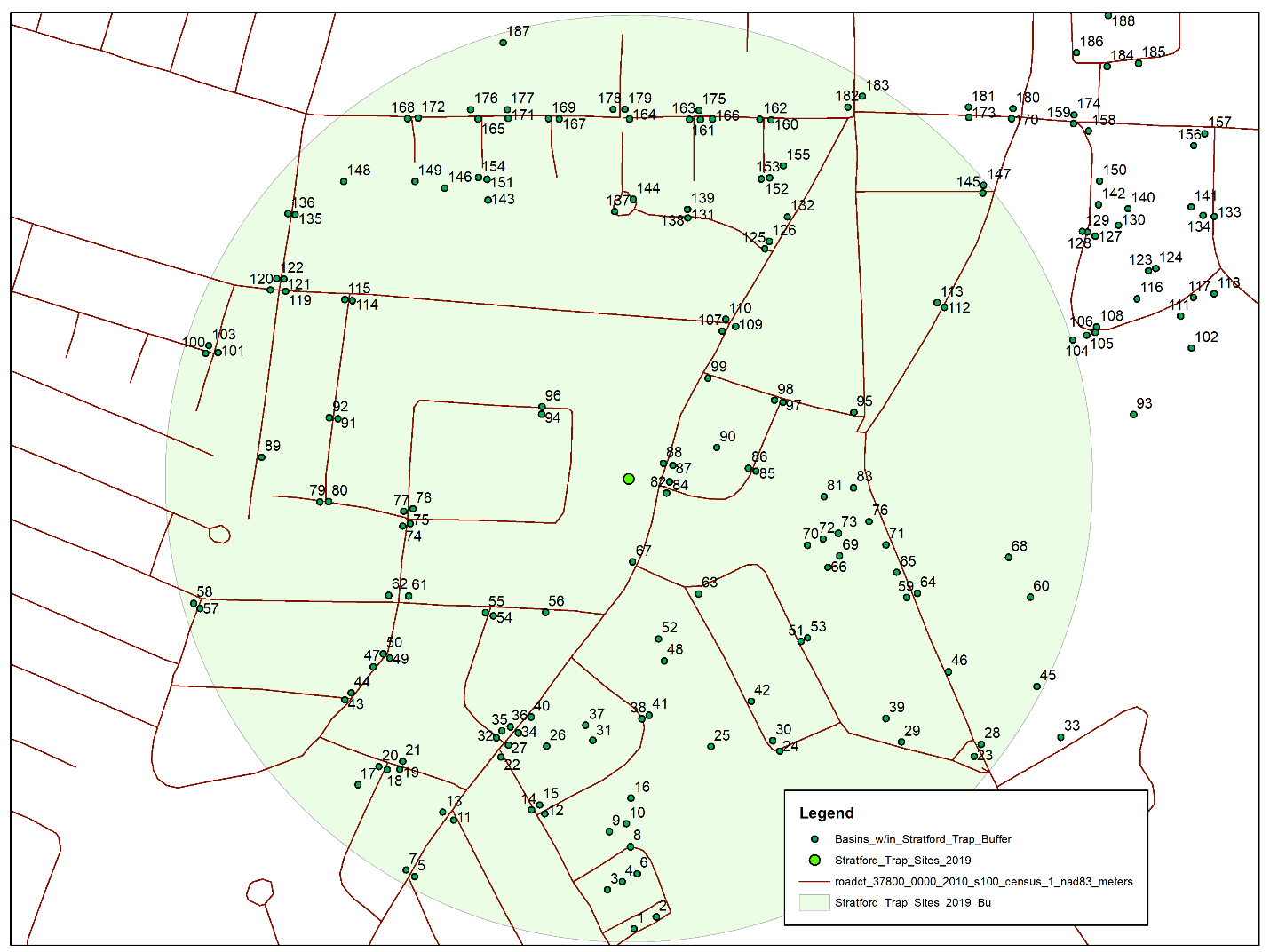


**S. Figure 7**. “Woodend” surveillance site in Stratford, Connecticut. The central bight green circle designates the placement of a single gravid and CO_2_-baited light trap for adult mosquito and West Nile virus surveillance. The light green shaded regions demarcates the 500m buffer surrounding the surveillance location. The small green points with labeled numbers designate the location of catch basins within the 500m buffer surrounding the surveillance location. Thin, brown lines designate roads. A portion of the “Cloverfield” 500m buffer overlaps with the “High Park” 500m buffer. Catch basins sampled in 2019 included: 18, 20, 43, 54, 55, 61, 62, 79, and one basin not listed in the GIS file (near 61 and 62).


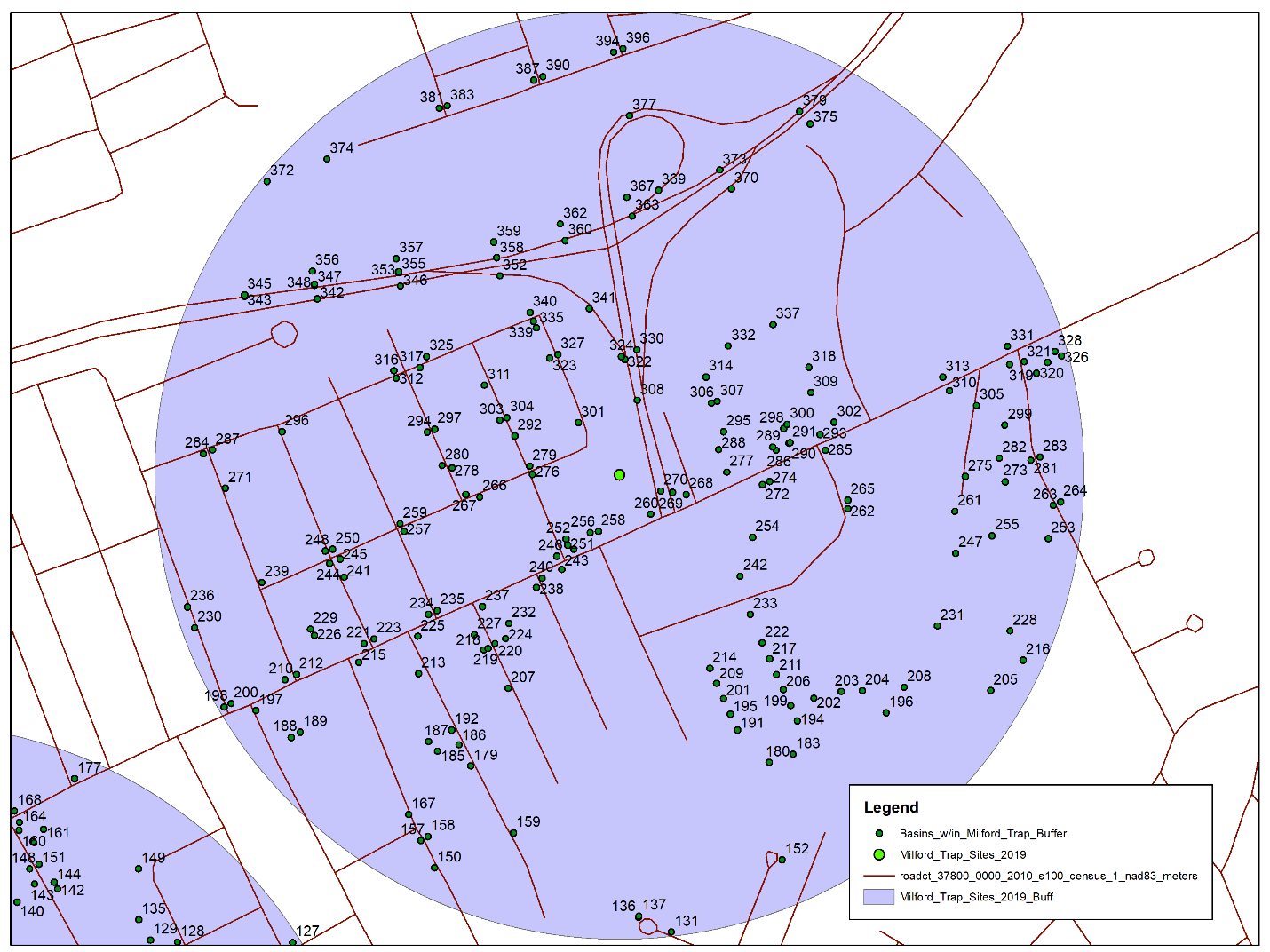


**S. Figure 8**. “Liberty Rock” surveillance site in Milford, Connecticut. The central bight green circle designates the placement of a single gravid and CO_2_-baited light trap for adult mosquito and West Nile virus surveillance. The light green shaded regions demarcates the 500m buffer surrounding the surveillance location. The small green points with labeled numbers designate the location of catch basins within the 500m buffer surrounding the surveillance location. Thin, brown lines designate roads. Catch basins sampled in 2019 included: 279, 303, 271, 250, and four basins just outside the 500m buffer (roads directly west of boundary).. Catch basins sampled in 2020 included: 179, 186, 192, 198, 207, 219, 220, 221, 223, 234, 235, 237, 244, 263, 266, 279, 319, 381, 387, and 390


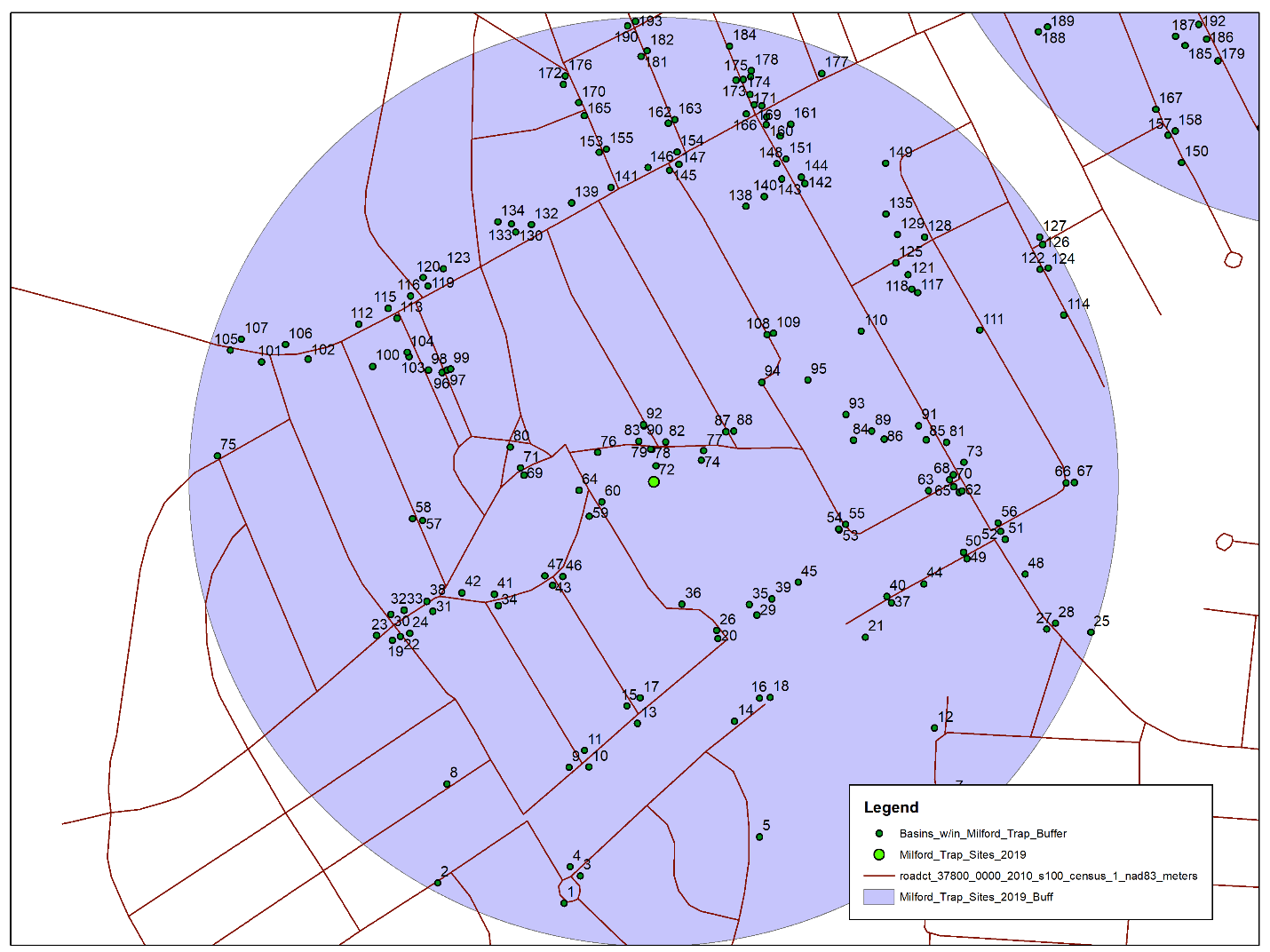


**S. Figure 9**. “Margaret Egan” surveillance site in Milford, Connecticut. The central bight green circle designates the placement of a single gravid and CO_2_-baited light trap for adult mosquito and West Nile virus surveillance. The light green shaded regions demarcates the 500m buffer surrounding the surveillance location. The small green points with labeled numbers designate the location of catch basins within the 500m buffer surrounding the surveillance location. Thin, brown lines designate roads. Catch basins sampled in 2020 included: 5, 31, 33, 38, 59, 73, 76, 77, 79, 81, 82, 99, 109, 119, 124, 125, 126, 127, 145, 149, and 172.


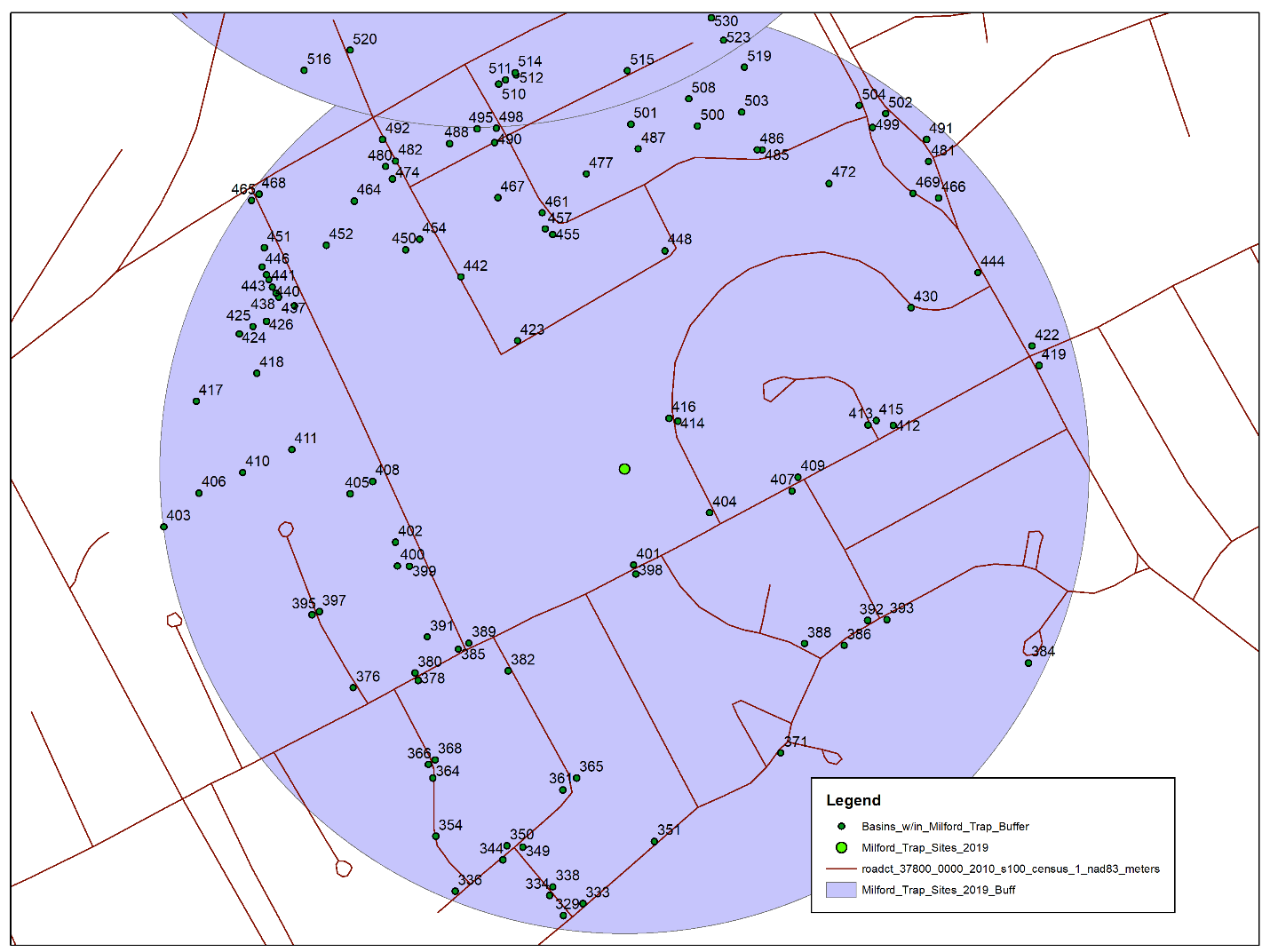


**S. Figure 10**. “Meadowside” surveillance site in Milford, Connecticut. The central bight green circle designates the placement of a single gravid and CO_2_-baited light trap for adult mosquito and West Nile virus surveillance. The light green shaded regions demarcates the 500m buffer surrounding the surveillance location. The small green points with labeled numbers designate the location of catch basins within the 500m buffer surrounding the surveillance location. Thin, brown lines designate roads. Catch basins sampled in 2019 included: 344, 349, 376, 382, 395, 397, 416 and three not listed in GIS file (one near 386 and two on cul-de-sac road directly outside western boundary of the 500m buffer). Catch basins sampled in 2020 included: 329, 338, 351, 380, 382, 384, 386, 395, 401, 419, and 422.


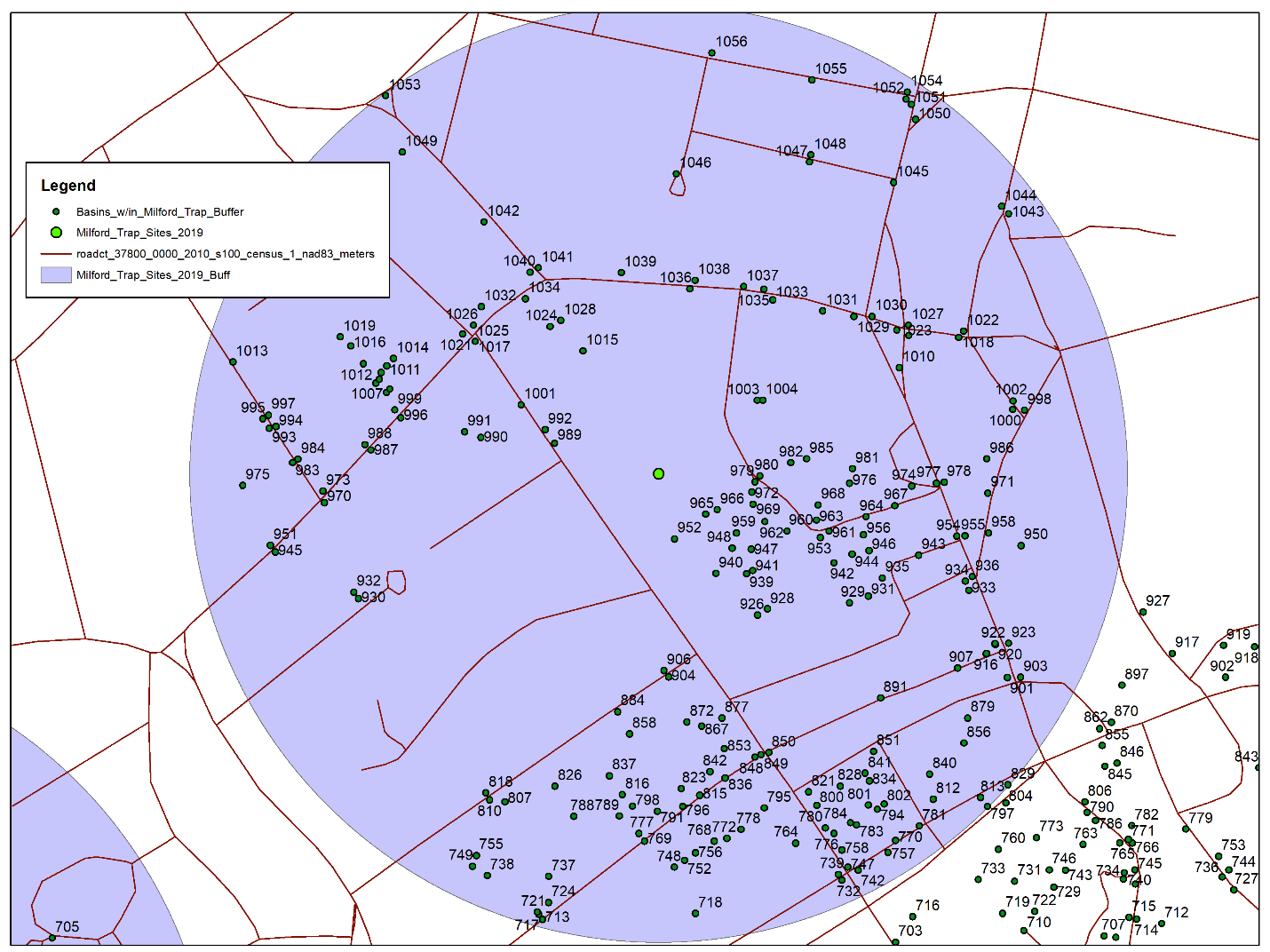


**S. Figure 11**. “Parsons Complex” surveillance site in Milford, Connecticut. The central bight green circle designates the placement of a single gravid and CO_2_-baited light trap for adult mosquito and West Nile virus surveillance. The light green shaded regions demarcates the 500m buffer surrounding the surveillance location. The small green points with labeled numbers designate the location of catch basins within the 500m buffer surrounding the surveillance location. Thin, brown lines designate roads. Catch basins sampled in 2020 included: 810, 850, 951, 955, 958, 967, 971, 978, 1000, 1001, 1002, 1004, 1022, 1038, 1042, 1047, 1048, and 1052.


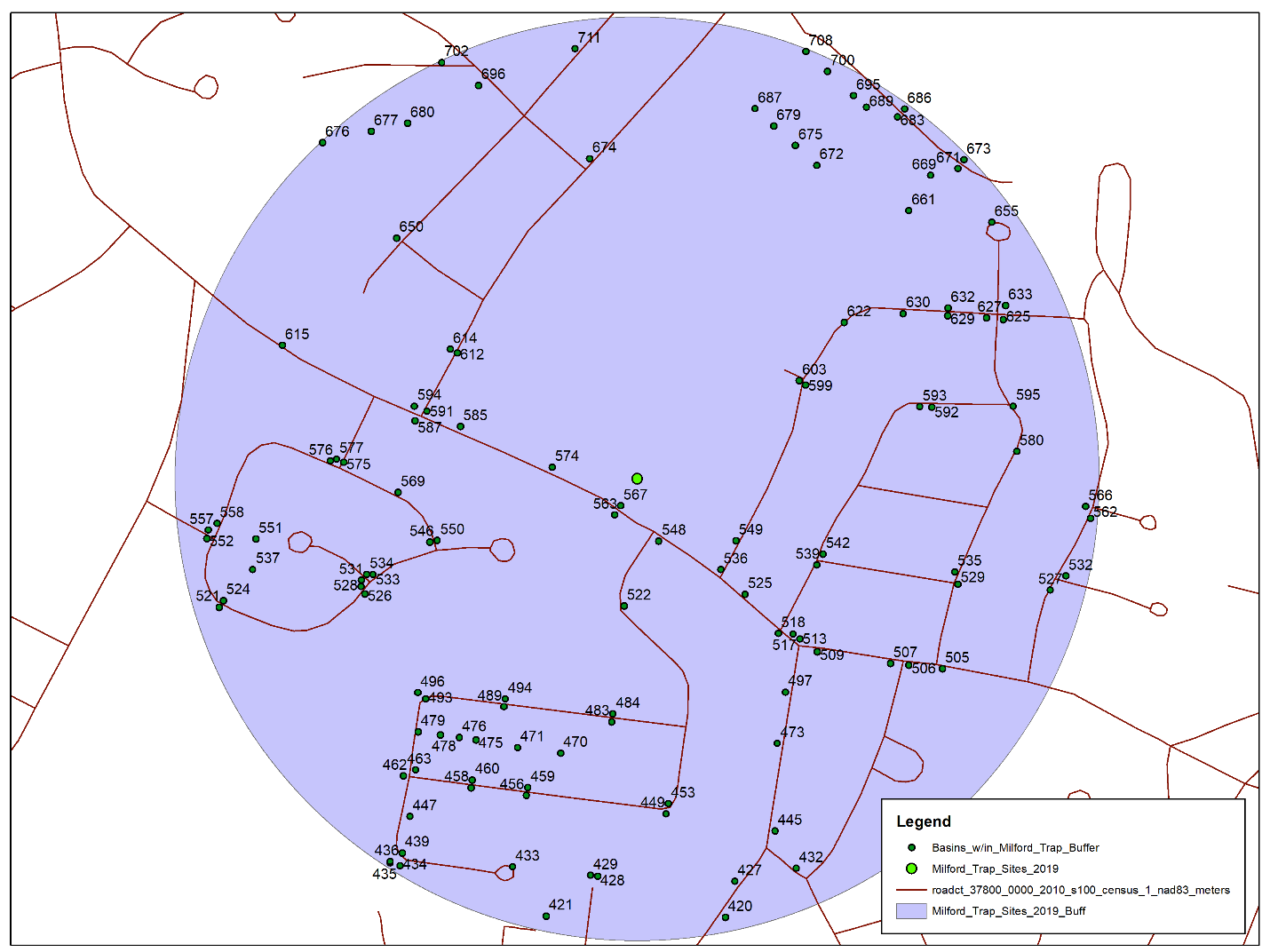


**S. Figure 12**. “Pond Point” surveillance site in Milford, Connecticut. The central bight green circle designates the placement of a single gravid and CO_2_-baited light trap for adult mosquito and West Nile virus surveillance. The light green shaded regions demarcates the 500m buffer surrounding the surveillance location. The small green points with labeled numbers designate the location of catch basins within the 500m buffer surrounding the surveillance location. Thin, brown lines designate roads. Catch basins sampled in 2019 included: 434, 435, 439, 456, 458, 463, 479, and one not listed in GIS file (near 433). Catch basins sampled in 2020 included: 433, 434, 435, 460, 463, 473, 522, 550, 557, 575, 595, and 671.


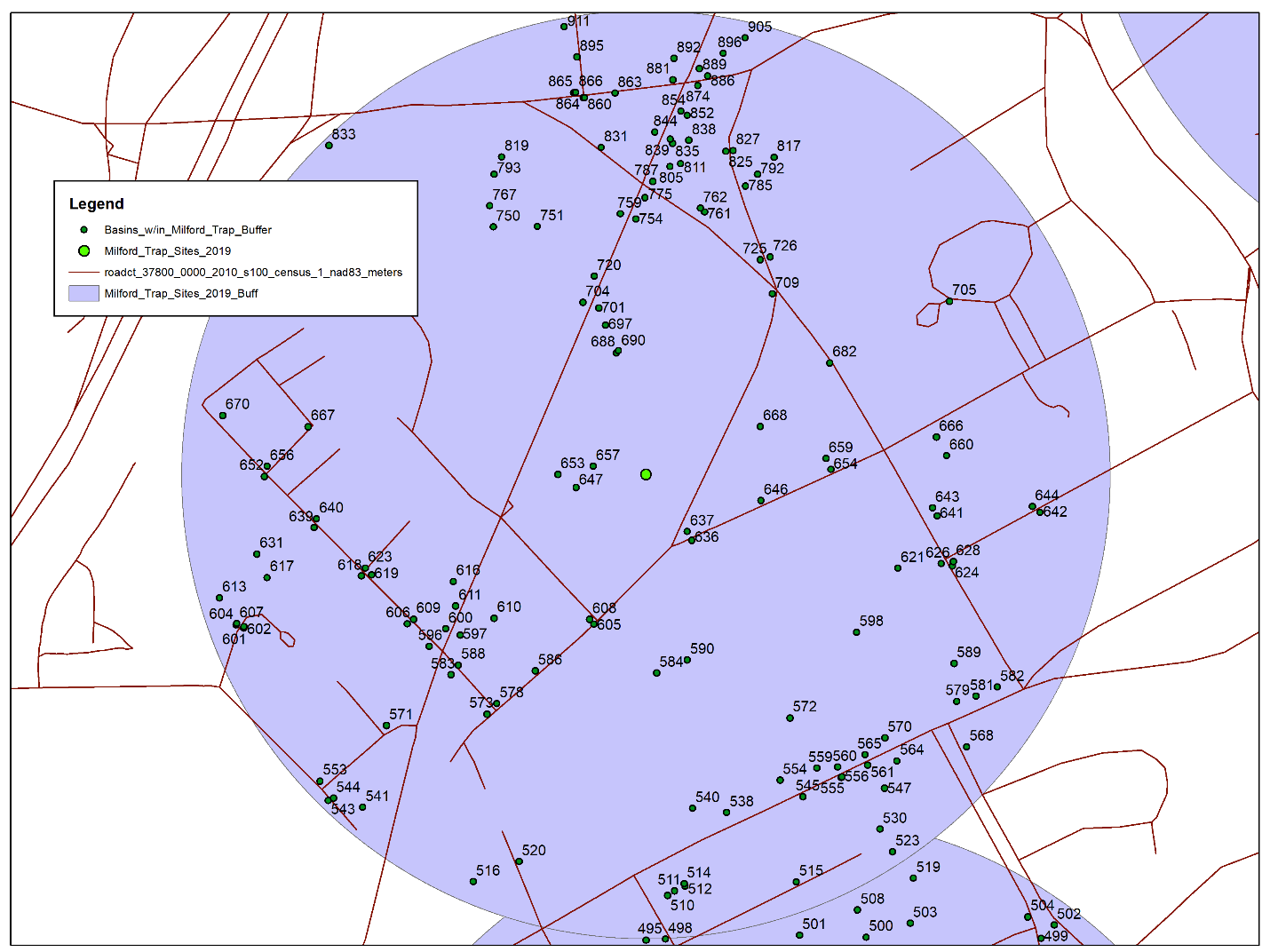


**S. Figure 13**. “Washington Field” surveillance site in Milford, Connecticut. The central bight green circle designates the placement of a single gravid and CO_2_-baited light trap for adult mosquito and West Nile virus surveillance. The light green shaded regions demarcates the 500m buffer surrounding the surveillance location. The small green points with labeled numbers designate the location of catch basins within the 500m buffer surrounding the surveillance location. Thin, brown lines designate roads. Catch basins sampled in 2020 included: 543, 544, 573, 583, 588, 601, 607, 619, 623, 624, 628, 654, and 667.


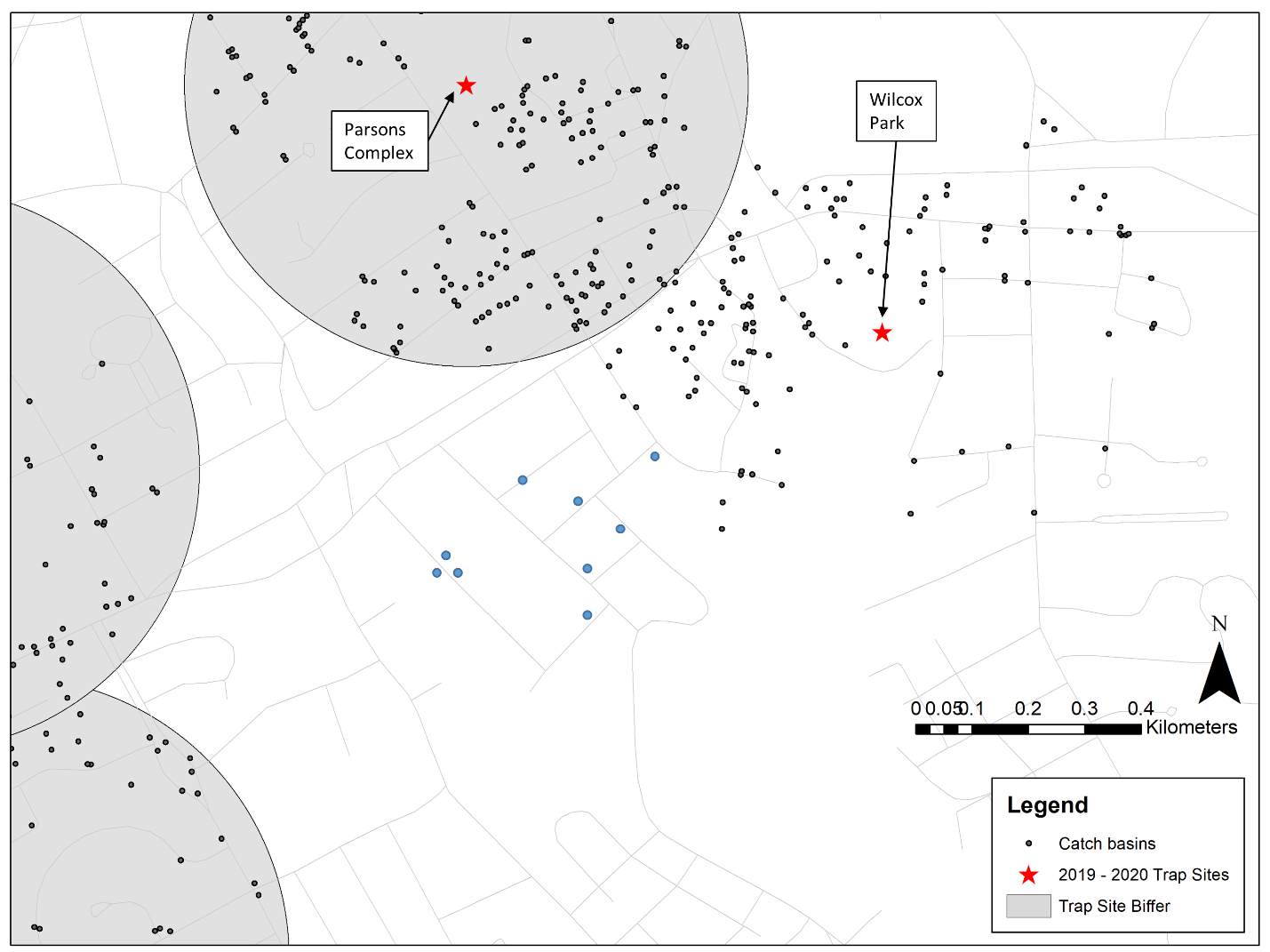


**S. Figure 14**. “Wilcox/Parsons Complex” catch basin surveillance site in Milford, Connecticut in 2019. Wilcox and Parsons Complex surveillance sites are indicated with red stars and labeled boxes. The Catch basins sampled in 2019 are drawn as blue circles.
