# Supplementary material for: The community‐wide effectiveness of municipal larval control programs for West Nile virus risk reduction in Co nnecticut, USA
Source: Pest Manag Sci. 2021 Aug 5;77(11):5186–201. doi: 10.1002/ps.6559 (PMC9291174; doi:10.1002/ps.6559)
Supplement: Supplementary file 3 — Appendix S3. Supporting Information [file PS-77-5186-s002.docx]

**Supporting information for:**

**Title.** The community-wide effectiveness of municipal larval control programs for West Nile virus risk reduction in Connecticut, United States

**Authors.** Joseph R. McMillan^1,2^, Christina A. Harden^3^, James C. Burtis^2,4^, Mallery I. Breban^5^, John J. Shepard^1^, Tanya A. Petruff^1^, Michael J. Misencik^1^, Angela B. Bransfield^1^, Joseph D. Poggi^2,5^, Laura C. Harrington^2,5^, Theodore G. Andreadis^1,2^, and Philip M. Armstrong^1,2^

**Author Affiliations.**

1. The Connecticut Agricultural Experiment Station, New Haven, CT
2. The Northeast Regional Center of Excellence in Vector-borne Diseases
3. Pennsylvania State University, State College, PA
4. Division of Vector-borne Diseases, Centers for Disease Control and Prevention, Fort Collins, CO
5. Cornell University, Ithaca NY
6. Yale University, New Haven, CT

**Corresponding Author**

Joseph R. McMillan

joseph.mcmillan@ct.gov

123 Huntington Street

New Haven, Connecticut 06511

Index

| Result | Page |
| --- | --- |
| Supporting Information | |
| 2020 Catch basin evaluations | 3 |
| GLMM Tables | |
| 2019 larval prevalence in catch basins | 4 |
| 2019 pupal prevalence in catch basins | 5 |
| 2019 *Cx. pipiens* collections, WNV positive pools, and WNV MIR from gravid traps | 6 |
| 2019 *Cx. pipiens* collections, WNV positive pools, and WNV MIR from gravid traps with corresponding basin sampling | 7 |
| 2019 *Cx. pipiens* collections from light traps | 8 |
| 2019 *Cx. pipiens* collections from light traps with corresponding basin sampling | 9 |
| 2020 larval prevalence and abundance in catch basins | 10 |
| 2020 pupal prevalence and abundance in catch basins | 11 |
| 2020 larval and pupal prevalence and abundance in catch basins random effects summaries | 12 |
| 2020 bioassay mortality results | 13 |
| 2020 *Cx. pipiens* collections, WNV positive pools, and WNV MIR from gravid traps with corresponding basin sampling | 14 |
| 2020 *Cx. pipiens* collections from light traps with corresponding basin sampling | 15 |

| Town | Location | Total Basins  (density, per km^2^) | Final basins | % (n) Basins evaluated  (% of total) | % (n) evaluated that were Surveyed in Field | % (n) field surveys with access restrictions | % (n) field surveys with no water | % (n) field surveys with restrictive Grates | % (n) field surveys removed due to sampling difficulties |
| --- | --- | --- | --- | --- | --- | --- | --- | --- | --- |
| Milford | Liberty Rock | 193 (245.9) | 20 | 69.9% (135) | 42.2% (57) | 8.8% (5) | 49.1% (28) | 8.8% (5) | 0 |
|  | Margaret Egan | 174 (221.7) | 18 | 26.4% (46) | 87% (40) | 15% (6) | 42.5% (17) | 0 | 0 |
|  | Meadowside | 111 (141.4) | 11 | 60.4% (67) | 59.7% (40) | 22.5% (9) | 25% (10) | 2.5% (1) | 0 |
|  | Parsons Complex | 208 (265.0) | 18 | 75% (156) | 52.6% (82) | 15.9% (13) | 46.3% (38) | 1.2% (1) | 13.4% (11) |
|  | Pond Point | 119 (151.6) | 12 | 34.4% (41) | 68.3% (28) | 19.5% (8) | 31.7% (13) | 0 | 0 |
|  | Washington Field | 131 (166.9) | 13 | 100% (131) | 39.7% (52) | 28.8% (15) | 46.2% (24) | 1.9% (1) | 5.8% (3) |
|  | ***Total*** | **936 (198.7)** | **92** | **61.5%** | **51.9%** | **18.7%** | **43.5%** | **2.7%** | **4.7%** |
| Stratford | Cloverfield | 98 (124.8) | 12 | 40.8% (40) | 82.5% (33) | 48.5% (16) | 39.4% (13) | 6.1% (2) | 3% (1) |
|  | High Park | 155 (197.5) | 16 | 38.7% (60) | 78.3% (47) | 14.9% (7) | 46.8% (22) | 2.1% (1) | 4.3% (2) |
|  | Historic District | 129 (164.3) | 12 | 40.3% (52) | 71.2% (37) | 0 | 43.2% (16) | 5.4% (2) | 0 |
|  | Long Brook | 108 (137.6) | 11 | 36.1% (39) | 71.8% (28) | 3.6% (1) | 46.4% (13) | 10.7% (3) | 0 |
|  | Stony Brook | 136 (173.2) | 14 | 23.5% (32) | 87.5% (28) | 7.1% (2) | 14.3% (4) | 3.6% (1) | 14.3% (4) |
|  | Woodend | 152 (193.6) | 15 | 33.6% (51) | 68.6% (35) | 14.35 (5) | 31.4% (11) | 8.6% (3) | 2.9% (1) |
|  | **Total** | **778 (165.2)** | **80** | **35.2%** | **75.9%** | **14.9%** | **36.1%** | **5.8%** | **3.8%** |

S. Table 1. Results from initial basin surveys in Milford and Stratford, Connecticut conducted between May 1^st^ and July 10^th^, 2020. Total basins represent all mapped basins within a 500m radius of the Location’s adult mosquito trapping location; basins evaluated represent all basins evaluated in order to achieve the desired ~10% of final basins; surveyed in the field represent all basins that visually appeared on the map to be on a small to medium sized road; access restrictions are defined as either any unsafe sampling environments, basin on private property, or any basin not found; no water represents the number of basins that were either dry or had only trace amounts of water during the initial field survey; restrictive grates indicate sampling devices could not fit through the grates to access the basin’s water source; removed due to sampling difficulties represent all basins initially designated as a final basin and then were removed after either repeated access issues, dry conditions, and/or inability to agree on the exact location of the basin.

| Variable | | Prevalence of any instar larvae | | | |
| --- | --- | --- | --- | --- | --- |
|  |  | Estimate | Std. Error | z-value | Pr(>\|z\|) |
| Intercept | | -2.76 | 1.17 | -2.36 | 0.02 |
| Town: Stratford | | 2.55 | 0.52 | 4.96 | 7.3e-7 |
| Climate | PRCP | --- | --- | --- | --- |
|  | TMP | 0.15 | 0.06 | 2.61 | 0.009 |
| Treatment Period | TRT.L1 | -0.42 | 0.68 | -0.62 | 0.54 |
|  | TRT.L2 | -0.96 | 0.74 | -1.29 | 0.20 |
|  | TRT.L3 | 0.85 | 0.56 | 1.52 | 0.13 |
|  | TRT.L4 | 0.21 | 0.48 | 0.45 | 0.66 |
| Town & Treatment Period interaction | STRAT: TRT.L0 | -0.51 | 1.13 | -0.45 | 0.66 |
|  | STRAT: TRT.L1 | -2.15 | 1.06 | -2.03 | 0.04 |
|  | STRAT: TRT.L2 | -3.15 | 1.01 | -3.13 | 0.002 |
|  | STRAT: TRT.L3 | -1.34 | 0.98 | -1.37 | 0.17 |
| Random Effect | | Type | Variance | Std. Dev. |  |
| Basin (n=67) | | Intercept | 3.21 | 1.80 |  |
| Week  (n=21) | | Intercept | 0.73 | 0.86 |  |

S. Table 2. Generalized linear mixed effect model (GLMM) results comparing prevalence of any instar larval detections in catch basins sampled in Milford and Stratford, Connecticut from June 10^th^ to October 29^th^, 2019. The GLMM utilized a binomial error distribution with Town as a fixed effect categorical effect (reference Milford), total weekly precipitation and average weekly temperature as continuous fixed effects, time since larvicide application as a categorical fixed effect, and basin and site of collection as crossed random effects. Dashed lines indicate removal of the variable improved AIC scores, and therefore was not included in the final GLMM.

| Variable | | Prevalence of pupae | | | |
| --- | --- | --- | --- | --- | --- |
|  |  | Estimate | Std. Error | z-value | Pr(>\|z\|) |
| Intercept | | -0.92 | 0.36 | -2.56 | 0.01 |
| Town: Stratford | | 1.45 | 0.41 | 3.50 | 0.0005 |
| Climate | PRCP | -0.09 | 0.06 | -1.65 | 0.10 |
|  | TMP | --- | --- | --- | --- |
| Treatment Period | TRT.L1 | --- | --- | --- | --- |
|  | TRT.L2 | --- | --- | --- | --- |
|  | TRT.L3 | --- | --- | --- | --- |
|  | TRT.L4 | --- | --- | --- | --- |
| Town by PRCP interaction | | -0.09 | 0.04 | -1.95 | 0.05 |
| Random Effect | | Type | Variance | Std. Dev. |  |
| Basin (n=67) | | Intercept | 2.10 | 1.45 |  |
| Week  (n=21) | | Intercept | 0.47 | 0.68 |  |

S. Table 3. Generalized linear mixed effect model (GLMM) results comparing prevalence of pupal detections in catch basins sampled in Milford and Stratford, Connecticut from June 10^th^ to October 29^th^, 2019. The GLMM utilized a binomial error distribution with Town as a fixed effect categorical effect (reference Milford), total weekly precipitation and average weekly temperature as continuous fixed effects, time since larvicide application as a categorical fixed effect, and basin and site of collection as crossed random effects. Dashed lines indicate removal of the variable improved AIC scores, and therefore was not included in the final GLMM.

| Variable | | Weekly Culex pipiens collections | | | | Weekly Culex pipiens WNV positive pools | | | | Weekly Culex pipiens WNV MIR | | | |
| --- | --- | --- | --- | --- | --- | --- | --- | --- | --- | --- | --- | --- | --- |
|  |  | Estimate | Std. Error | z-value | Pr(>\|z\|) | Estimate | Std. Error | z-value | Pr(>\|z\|) | Estimate | Std. Error | t-value | Pr(>\|z\|) |
| Intercept | | -2.88 | 0.78 | -3.70 | 0.0002 | -3.29 | 0.74 | -4.43 | 9.4e-6 | 0.38 | 0.17 | 2.21 | 0.02 |
| Town: Stratford | | --- | --- | --- | --- | --- | --- | --- | --- | --- | --- | --- | --- |
| Climate | PRCP | --- | --- | --- | --- | --- | --- | --- | --- | --- | --- | --- | --- |
|  | TEMP | 0.30 | 0.04 | 8.31 | < 2e-16 | --- | --- | --- | --- | --- | --- | --- | --- |
| Treatment Period | TRT.L1 | --- | --- | --- | --- | -1.21 | 0.83 | -1.45 | 0.14 | -0.91 | 0.35 | -2.64 | 0.008 |
|  | TRT.L2 | --- | --- | --- | --- | -19.8 | 9719.9 | -0.002 | 0.99 | -0.85 | 0.33 | -2.58 | 0.01 |
|  | TRT.L3 | --- | --- | --- | --- | 0.05 | 0.61 | 0.08 | 0.94 | -0.40 | 0.33 | -1.19 | 0.23 |
|  | TRT.L4 | --- | --- | --- | --- | -0.76 | 0.54 | -1.41 | 0.16 | -0.40 | 0.29 | -1.40 | 0.16 |
|  | | | | | | | | | | | | | |
| Random effects | | Type | Variance | Std. Dev |  | Type | Variance | Std. Dev |  | Type | Variance | Std. Dev |  |
| Week of collection (n=20) | | Intercept | 0.32 | 0.57 |  | Intercept | 4.15 | 2.04 |  | Intercept | 0.39 | 0.62 |  |
| Site (n=16) | | Intercept | 0.07 | 0.26 |  | Intercept | --- | --- |  | Intercept | --- | --- |  |

S. Table 4. Generalized linear mixed effect model (GLMM) results comparing *Culex pipiens* collections in gravid traps, number of West Nile virus (WNV) positive pools, and WNV minimum infection rates (MIR, per 1,000 tested individuals) between Milford and Stratford, Connecticut from June 10^th^ to October 29^th^, 2019. All GLMMs utilized all trapping locations and site and week as crossed random effects. GLMMs for collections utilized a negative-binomial error distribution; GLMMs for number of positive pools utilized a Poisson-error distribution with a log transformed intercept offset for the number of pools tested; WNV MIRs utilized a Gaussian error distribution with the response variable log+1 transformed and a log transformed intercept offset for the number of pools tested. Dashed lines indicate removal of the variable improved AIC scores, and therefore was not included in the final GLMM.

| Variable | | Weekly Culex pipiens collections | | | | Weekly Culex pipiens WNV positive pools | | | | Weekly Culex pipiens WNV MIR | | | |
| --- | --- | --- | --- | --- | --- | --- | --- | --- | --- | --- | --- | --- | --- |
|  |  | Estimate | Std. Error | z-value | Pr(>\|z\|) | Estimate | Std. Error | z-value | Pr(>\|z\|) | Estimate | Std. Error | t-value | Pr(>\|z\|) |
| Intercept | | -3.22 | 0.96 | -3.35 | 0.0008 | -2.63 | 0.65 | -4.01 | 6e-5 | -0.01 | 0.23 | -0.05 | 0.96 |
| Town: Stratford | | --- | --- | --- | --- | --- | --- | --- | --- | --- | --- | --- | --- |
| Climate | PRCP | --- | --- | --- | --- | -0.25 | 0.18 | -1.41 | 0.16 | -0.09 | 0.05 | -1.76 | 0.08 |
|  | TEMP | 0.32 | 0.04 | 7.54 | 4.8e-14 | --- | --- | --- | --- | --- | --- | --- | --- |
| Metrics of mosquito occupancy | PRV Eggs | --- | --- | --- | --- | --- | --- | --- | --- | --- | --- | --- | --- |
|  | Sum Eggs | --- | --- | --- | --- | --- | --- | --- | --- | --- | --- | --- | --- |
|  | PRV Larvae | --- | --- | --- | --- | --- | --- | --- | --- | --- | --- | --- | --- |
|  | Sum Larvae | --- | --- | --- | --- | --- | --- | --- | --- | --- | --- | --- | --- |
|  | PRV pupae | --- | --- | --- | --- | --- | --- | --- | --- | --- | --- | --- | --- |
|  | Sum Pupae | -0.07 | 0.04 | -1.55 | 0.12 | --- | --- | --- | --- | --- | --- | --- | --- |
| Treatment Period | TRT.L1 | --- | --- | --- | --- | --- | --- | --- | --- | --- | --- | --- | --- |
|  | TRT.L2 | --- | --- | --- | --- | --- | --- | --- | --- | --- | --- | --- | --- |
|  | TRT.L3 | --- | --- | --- | --- | --- | --- | --- | --- | --- | --- | --- | --- |
|  | TRT.L4 | --- | --- | --- | --- | --- | --- | --- | --- | --- | --- | --- | --- |
|  | | | | | | | | | | | | | |
| Random effects | | Type | Variance | Std. Dev |  | Type | Variance | Std. Dev |  | Type | Variance | Std. Dev |  |
| Week of collection (n=20) | | Intercept | 0.43 | 0.65 |  | Intercept | 2.20 | 1.49 |  | Intercept | 0.37 | 0.61 |  |
| Site (n=8) | | Intercept | 0.09 | 0.30 |  | Intercept | --- | --- |  | Intercept | --- | --- |  |

S. Table 5. Generalized linear mixed effect model (GLMM) results comparing *Culex pipiens* collections in gravid traps, number of West Nile virus (WNV) positive pools, and WNV minimum infection rates (MIR, per 1,000 tested individuals) between Milford and Stratford, Connecticut from June 10^th^ to October 29^th^, 2019. All GLMMs utilized a subset of trapping locations with accompanying catch basin information and site and week as crossed random effects. GLMMs for collections utilized a negative-binomial error distribution; GLMMs for number of positive pools utilized a Poisson-error distribution with a log+1 transformed intercept offset for the number of pools tested; WNV MIRs utilized a Gaussian error distribution with the response variable log+1 transformed and a log+1 transformed intercept offset for the number of pools tested. Dashed lines indicate removal of the variable improved AIC scores, and therefore was not included in the final GLMM. Exclamation points indicate that only a single metric of larval and pupal occupancy was considered when defining the best fit model (i.e., the best fitting variable was included in the final model.

| Variable | | Weekly Culex pipiens collections | | | |
| --- | --- | --- | --- | --- | --- |
|  |  | Estimate | Std. Error | z-value | Pr(>\|z\|) |
| Intercept | | -3.47 | 1.18 | -2.93 | 0.003 |
| Town: Stratford | | 0.93 | 0.42 | 2.25 | 0.02 |
| Climate | PRCP | --- | --- | --- | --- |
|  | TEMP | 0.21 | 0.05 | 4.05 | 5.2e-5 |
| Treatment Period | TRT.L1 | --- | --- | --- | --- |
|  | TRT.L2 | --- | --- | --- | --- |
|  | TRT.L3 | --- | --- | --- | --- |
|  | TRT.L4 | --- | --- | --- | --- |
| Random effects | | Type | Variance | Std. Dev |  |
| Week of collection (n=19) | | Intercept | 0.52 | 0.72 |  |
| Site (n=16) | | Intercept | 0.31 | 0.56 |  |

S. Table 6. Generalized linear mixed effect model (GLMM) results comparing *Culex pipiens* collections in CO_2_-baited ground level light traps between Milford and Stratford, Connecticut from June 10^th^ to October 29^th^, 2019. The GLMM utilized all light trap locations, site and week as crossed random effects, and a negative binomial error distribution. Dashed lines indicate removal of the variable improved AIC scores, and therefore was not included in the final GLMM.

| Variable | | Weekly Culex pipiens collections | | | |
| --- | --- | --- | --- | --- | --- |
|  |  | Estimate | Std. Error | z-value | Pr(>\|z\|) |
| Intercept | | -7.15 | 1.62 | -4.42 | 1e-5 |
| Town: Stratford | | 1.63 | 0.82 | 2.00 | 0.05 |
| Climate | PRCP |  |  |  |  |
|  | TEMP | 0.32 | 0.07 | 4.80 | 1.6e-6 |
| Metrics of mosquito occupancy | PRV Eggs | --- | --- | --- | --- |
|  | Sum Eggs | --- | --- | --- | --- |
|  | PRV Larvae | --- | --- | --- | --- |
|  | Sum Larvae | --- | --- | --- | --- |
|  | PRV pupae | --- | --- | --- | --- |
|  | Sum Pupae | --- | --- | --- | --- |
| Treatment Period | TRT.L1 | --- | --- | --- | --- |
|  | TRT.L2 | --- | --- | --- | --- |
|  | TRT.L3 | --- | --- | --- | --- |
|  | TRT.L4 | --- | --- | --- | --- |
| Random effects | | Type | Variance | Std. Dev | |
| Week of collection (n=20) | | Intercept | 0.74 | 0.86 | |
| Site (n=8) | | Intercept | 1.16 | 1.08 | |

S. Table 7. Generalized linear mixed effect model (GLMM) results comparing *Culex pipiens* collections in CO_2_-baited ground level light traps between Milford and Stratford, Connecticut from June 10^th^ to October 29^th^, 2019. The GLMM utilized a subset of trapping locations with accompanying catch basin information, site and week as crossed random effects, and a negative binomial error distribution. Dashed lines indicate removal of the variable improved AIC scores, and therefore was not included in the final GLMM.

| Variable: Main Effects | | Prevalence of IV instar larvae | | | | Number of IV instar larvae | | | |
| --- | --- | --- | --- | --- | --- | --- | --- | --- | --- |
|  |  | Estimate | Std. Error | z-value | Pr(>\|z\|) | Estimate | Std. Error | z-value | Pr(>\|z\|) |
| Intercept | | -1.59 | 0.36 | -4.41 | 1.1e-5 | -0.33 | 0.37 | -0.88 | 0.38 |
| Town: Stratford | | 1.50 | 0.34 | 4.43 | 9.4e-6 | 1.11 | 0.36 | 3.05 | 0.002 |
| PRCP | | -0.01 | 0.007 | -1.91 | 0.06 | --- | --- | --- | --- |
| TRT Period | L1 | -0.85 | 0.31 | -2.74 | 0.006 | -1.07 | 0.29 | -3.71 | 0.0002 |
|  | L2 | -0.47 | 0.37 | -1.29 | 0.20 | -0.85 | 0.35 | -2.42 | 0.02 |
|  | L3 | 0.36 | 0.34 | 1.05 | 0.30 | -0.40 | 0.34 | -1.19 | 0.24 |
|  | L4 | 0.69 | 0.34 | 1.99 | 0.05 | -0.36 | 0.34 | -1.08 | 0.28 |
|  | | | | | |  |  |  |  |
| Variable: Interactions | | Df | AIC | LRT | Pr(Chi) | Df | AIC | LRT | Pr(Chi) |
| None | |  | 2788.9 |  |  |  | 10479 |  |  |
| Town*TRT | | 4 | 2802.5 | 21.57 | 0.0002 | 4 | 10480 | 8.92 | 0.06 |
|  | |  |  |  |  |  |  |  |  |
| Random Effects | | Type | Variance | Std. Dev. | | Variance | | Std. Dev. | |
| Basin (n=186) | | Intercept | 3.57 | 1.89 | | 4.37 | | 2.09 | |
| Week (n=20) | | Intercept | 0.89 | 0.95 | | 1.30 | | 1.14 | |

S. Table 8. Generalized linear mixed effect model (GLMM) results comparing prevalence of larval detections and the number of IV instar larval collections in catch basins sampled in Milford and Stratford, Connecticut from June 1^st^ to October 16^th^, 2020. The prevalence GLMM utilized a binomial error distribution with basin and site of collection as crossed random effects. The collection GLMM utilized a negative binomial error distribution with basin and site of collection as crossed random effects. The best fitting variables by AIC are shown. Information listed for Interaction terms display the results from a Drop 1 χ^2^ test. Dashed lines indicate the variable did not improve AIC and was therefore removed from the model.

| Variable: Main Effects | | Prevalence of pupae | | | | Number of pupae | | | |
| --- | --- | --- | --- | --- | --- | --- | --- | --- | --- |
|  |  | Estimate | Std. Error | z-value | Pr(>\|z\|) | Estimate | Std. Error | z-value | Pr(>\|z\|) |
| Intercept | | -1.98 | 0.33 | -5.96 | 2.6e-9 | -0.76 | 0.46 | -1.66 | 0.10 |
| Town: Stratford | | 1.24 | 0.32 | 3.86 | 0.0001 | 0.91 | 0.43 | 2.13 | 0.03 |
| PRCP | | -0.02 | 0.006 | -3.10 | 0.002 | -0.07 | 0.02 | -4.09 | 4.3e-5 |
| TRT Period | L1 | -0.61 | 0.31 | -1.96 | 0.05 | -1.74 | 0.56 | -3.26 | 0.001 |
|  | L2 | -0.81 | 0.38 | -2.16 | 0.03 | -0.41 | 0.56 | -0.74 | 0.46 |
|  | L3 | 0.26 | 0.34 | 0.75 | 0.45 | 0.37 | 0.82 | 0.45 | 0.65 |
|  | L4 | 0.69 | 0.37 | 1.89 | 0.06 | 0.11 | 0.52 | 0.22 | 0.83 |
| Variable: Interactions | | Df | AIC | LRT | Pr(Chi) | Df | AIC | LRT | Pr(Chi) |
| None | |  | 2607 |  |  |  | 7378.8 |  |  |
| Town*TRT | | 4 | 2616.0 | 16.8 | 0.002 | 4 | 7397.6 | 26.8 | 2.1e-5 |
| Town*PRCP | | --- | --- | --- | --- | 1 | 7386.3 | 9.54 | 0.002 |
| TRT*PRCP | | --- | --- | --- | --- | 4 | 7384.5 | 13.7 | 0.008 |
|  | |  |  |  |  |  |  |  |  |
| Random Effects | | Type | Variance | Std. Dev. | | Variance | | Std. Dev. | |
| Basin (n=186) | | Intercept | 2.98 | 1.72 | | 4.81 | | 2.19 | |
| Week (n=20) | | Intercept | 0.6 | 0.81 | | 1.16 | | 1.08 | |

S. Table 9. Generalized linear mixed effect model (GLMM) results comparing prevalence of pupal detections and the number of pupal collections in catch basins sampled in Milford and Stratford, Connecticut from June 1^st^ to October 16^th^, 2020. The prevalence GLMM utilized a binomial error distribution with basin and site of collection as crossed random effects. The collection GLMM utilized a negative binomial error distribution with basin and site of collection as crossed random effects. The best fitting variables by AIC are shown. Information listed for Interaction terms display the results from a Drop 1 χ^2^ test. Dashed lines indicate the variable did not improve AIC and was therefore removed from the model.

| Town | Basin Sampling Zone | IV instar larvae collections: catch basin random effects | | Pupal collections: catch basin random effects | |
| --- | --- | --- | --- | --- | --- |
|  |  | Positive random effects 95%CI >0 | Negative random effects 95%CI <0 | Positive random effects 95%CI >0 | Negative random effects 95%CI <0 |
| Milford | Liberty Rock (n=20) | 4 | 4 | 4 | 1 |
|  | Margaret Egan (n=18) | 4 | 2 | 5 | 0 |
|  | Meadowside (n=11) | 5 | 1 | 3 | 4 |
|  | Parsons Complex (n=18) | 9 | 4 | 7 | 2 |
|  | Pond Point (n=12) | 3 | 2 | 7 | 2 |
|  | Washington Field (n=13) | 2 | 5 | 0 | 3 |
|  | **Total (n=92)** | **27** | **18** | **26** | **12** |
| Stratford | Cloverfield (n=12) | 1 | 6 | 0 | 7 |
|  | High Park (n=16) | 4 | 2 | 5 | 1 |
|  | Historic District (n=12) | 6 | 2 | 7 | 2 |
|  | Long Brook (n=11) | 2 | 3 | 1 | 3 |
|  | Stony Brook (n=14) | 4 | 3 | 4 | 2 |
|  | Woodend (n=13) | 6 | 2 | 6 | 2 |
|  | **Total (n=80)** | **23** | **18** | **23** | **17** |

S. Table 10. Summary random intercept effect estimates from a generalized linear mixed effect model (GLMM) comparing mosquito IV instar larval and pupal collections in catch basins between Milford and Stratford, Connecticut from June 1^st^ to October 16^th^, 2020. GLMMs utilized a negative-binomial error distribution with Town as a fixed effect and week and catch basin as crossed random effects. There are no statistical differences in the proportion of basins with positive or negative intercept estimates between the two towns (evaluated using a two-sided proportion test).

| Variable: Main Effects | | Mortality | | | |
| --- | --- | --- | --- | --- | --- |
|  |  | Estimate | Std. Error | z-value | Pr(>\|z\|) |
| Intercept | | -0.67 | 0.31 | -2.17 | 0.03 |
| Town: Stratford | | -1.65 | 0.36 | -4.58 | 4.7e-6 |
| PRCP | | -0.06 | 0.02 | -3.48 | 0.0005 |
| TRT Period | L1 | 0.04 | 0.62 | 0.06 | 0.95 |
|  | L2 | 1.13 | 0.58 | 1.95 | 0.05 |
|  | L3 | 0.08 | 0.59 | 0.14 | 0.89 |
|  | L4 | -0.22 | 0.49 | -0.44 | 0.66 |
| Variable: Interactions | | Df | AIC | LRT | Pr(Chi) |
| None | |  | 1029.5 |  |  |
| Town*PRCP | | 1 | 1036.0 | 8.46 | 0.004 |
| TRT*PRCP | | 4 | 1032.1 | 10.5 | 0.03 |
| Random Effects | | Type | Variance | Std. Dev. | |
| Basin (n=186) | | Intercept | 0.64 | 0.80 | |
| Week (n=20) | | Intercept | 0.14 | 0.37 | |

S. Table 11. Generalized linear mixed effect model (GLMM) results comparing water sample mortality estimates from catch basins sampled in Milford and Stratford, Connecticut from June 1^st^ to October 16^th^, 2020: samples were limited to those tested when zero larvae and pupae were detected. The GLMM utilized a binomial error distribution with basin and site of collection as crossed random effects. Information listed for Interaction terms display the results from a Drop 1 χ^2^ test.

| Variable | | Culex pipiens collections | | | | Weekly Culex pipiens WNV positive pools | | | | Weekly Culex pipiens WNV MIR | | | |
| --- | --- | --- | --- | --- | --- | --- | --- | --- | --- | --- | --- | --- | --- |
|  |  | Estimate | Std. Error | z-value | Pr(>\|z\|) | Estimate | Std. Error | z-value | Pr(>\|z\|) | Estimate | Std. Error | t-value | Pr(>\|z\|) |
| Intercept | | 4.23 | 0.20 | 21.2 | <2e-16 | -4.36 | 0.65 | -6.72 | 1.8e-11 | -0.79 | 0.13 | -5.93 | 3.1e-9 |
| Town: Stratford | | --- | --- | --- | --- | 0.83 | 0.29 | 2.83 | 0.005 | 0.3 | 0.13 | 2.31 | 0.02 |
| Climate | PRCP | --- | --- | --- | --- | --- | --- | --- | --- | --- | --- | --- | --- |
|  | TEMP | 0.22 | 0.04 | 5.59 | 2.3e-8 | 0.26 | 0.16 | 1.62 | 0.11 | 0.003 | 0.001 | 2.63 | 0.009 |
| Metrics of pupae in catch basins^!^ | Avg. pupae | --- | --- | --- | --- | --- | --- | --- | --- | --- | --- | --- | --- |
|  | N basins pupae detected | --- | --- | --- | --- | --- | --- | --- | --- | --- | --- | --- | --- |
|  | Total Pupae | 0.003 | 0.001 | 2.62 | 0.009 | --- | --- | --- | --- | --- | --- | --- | --- |
|  | Total CXP Pupae | --- | --- | --- | --- | --- | --- | --- | --- | --- | --- | --- | --- |
| Metrics of IV instar larvae in catch basins^!^ | Avg. IV instar larvae | --- | --- | --- | --- | --- | --- | --- | --- | --- | --- | --- | --- |
|  | N basins larvae detected | -0.08 | 0.03 | -2.69 | 0.007 | --- | --- | --- | --- | --- | --- | --- | --- |
|  | Total IV larvae | --- | --- | --- | --- | --- | --- | --- | --- | --- | --- | --- | --- |
|  | Total CXP larvae | --- | --- | --- | --- | --- | --- | --- | --- | --- | --- | --- | --- |
| Mortality Estimate | | --- | --- | --- | --- | --- | --- | --- | --- | --- | --- | --- | --- |
| Treatment Period | L1 | --- | --- | --- | --- | --- | --- | --- | --- | --- | --- | --- | --- |
|  | L2 | --- | --- | --- | --- | --- | --- | --- | --- | --- | --- | --- | --- |
|  | L3 | --- | --- | --- | --- | --- | --- | --- | --- | --- | --- | --- | --- |
|  | L4 | --- | --- | --- | --- | --- | --- | --- | --- | --- | --- | --- | --- |
| Random effects | | Type | Variance | Std. Dev |  | Type | Variance | Std. Dev |  | Type | Variance | Std. Dev |  |
| Week of collection (n=20) | | Intercept | 0.31 | 0.56 |  | Intercept | 2.49 | 1.58 |  | Intercept | 0.19 | 0.43 |  |
| Site (n=12) | | Intercept | 0.04 | 0.20 |  | Intercept | --- | --- |  | Intercept | --- | --- |  |

S. Table 12. Generalized linear mixed effect model (GLMM) results comparing *Culex pipiens* collections in gravid traps, number of West Nile virus (WNV) positive pools, and WNV minimum infection rates (MIR, per 1,000 tested individuals) between Milford and Stratford, Connecticut from June 1^st^ to October 16^th^, 2020. All GLMMs utilized site and week as crossed random effects. GLMMs for collections utilized a negative-binomial error distribution; GLMMs for number of positive pools utilized a Poisson-error distribution with a log+1 transformed intercept offset for the number of pools tested; WNV MIRs utilized a Gaussian error distribution with the response variable log+1 transformed and a log+1 transformed intercept offset for the number of pools tested. Dashed lines indicate removal of the variable improved AIC scores, and therefore was not included in the final GLMM. Exclamation points indicate that only a single metric of larval and pupal occupancy was considered when defining the best fit model (i.e., the best fitting variable was included in the final model.

| Variable | |  |  |  |  |
| --- | --- | --- | --- | --- | --- |
|  |  | Culex pipiens collection | | | |
|  |  | Estimate | Std. Error | z-value | Pr(>\|z\|) |
| Intercept | | -0.61 | 0.53 | -1.15 | 0.25 |
| Town: Stratford | | 1.86 | 0.54 | 3.41 | 0.0007 |
| Climate | PRCP | --- | **---** | --- | --- |
|  | TEMP | --- | --- | --- | --- |
| Metrics of pupae in catch basins^!^ | Avg. pupae | --- | --- | --- | --- |
|  | N basins pupae detected | --- | --- | --- | --- |
|  | Total Pupae | --- | --- | --- | --- |
|  | Total CXP Pupae | --- | --- | --- | --- |
| Metrics of IV instar larvae in catch basins^!^ | Avg. IV instar larvae | --- | --- | --- | --- |
|  | N basins larvae detected | 0.11 | 0.05 | 2.13 | 0.03 |
|  | Total IV larvae | --- | --- | --- | --- |
|  | Total CXP larvae | --- | --- | --- | --- |
| Mortality Estimate | | --- | --- | --- | --- |
| Treatment Period | L1 | 0.19 | 0.48 | 0.40 | 0.69 |
|  | L2 | 0.08 | 0.51 | 0.15 | 0.88 |
|  | L3 | 0.40 | 0.48 | 0.82 | 0.41 |
|  | L4 | -1.21 | 0.46 | -2.63 | 0.009 |
| Random effects | | Type | Variance | Std. Dev |  |
| Week of collection (n=20) | | Intercept | 1.03 | 1.03 |  |
| Site (n=12) | | Intercept | 0.64 | 0.80 |  |
| Zero-inflation parameter | | Estimate | Std. error | z-value | PR(>\|z\|) |
|  |  | -1.69 | 0.48 | -3.52 | 0.0004 |

S. Table 13. Generalized linear mixed effect model (GLMM) results comparing *Culex pipiens* collections in CO_2_-baited, ground level light traps between Milford and Stratford, Connecticut from June 1^st^ to October 16^th^, 2020. The GLMM utilized a negative-binomial error distribution, accounted for zero-inflation, and utilized week and site of collection as crossed random effects. Metrics of West Nile virus in light traps were not compared due to a dearth of sample points to compare between the two towns.
